# Mapping the substrate landscape of protein phosphatase 2A catalytic subunit PPP2CA

**DOI:** 10.1016/j.isci.2024.109302

**Published:** 2024-02-19

**Authors:** Abigail Brewer, Gajanan Sathe, Billie E. Pflug, Rosemary G. Clarke, Thomas J. Macartney, Gopal P. Sapkota

**Affiliations:** 1 Medical Research Council (MRC) Protein Phosphorylation & Ubiquitylation Unit, School of Life Sciences, University of Dundee, Dundee DD1 5EH, UK

**Keywords:** Enzymology, Protein, Properties of biomolecules, Proteomics

## Abstract

Protein phosphatase 2A (PP2A) is an essential Ser/Thr phosphatase. The PP2A holoenzyme complex comprises a scaffolding (A), regulatory (B), and catalytic (C) subunit, with PPP2CA being the principal catalytic subunit. The full scope of PP2A substrates in cells remains to be defined. To address this, we employed dTAG proteolysis-targeting chimeras to efficiently and selectively degrade dTAG-PPP2CA in homozygous knock-in HEK293 cells. Unbiased global phospho-proteomics identified 2,204 proteins with significantly increased phosphorylation upon dTAG-PPP2CA degradation, implicating them as potential PPP2CA substrates. A vast majority of these are novel. Bioinformatic analyses revealed involvement of the potential PPP2CA substrates in spliceosome function, cell cycle, RNA transport, and ubiquitin-mediated proteolysis. We identify a pSP/pTP motif as a predominant target for PPP2CA and confirm some of our phospho-proteomic data with immunoblotting. We provide an in-depth atlas of potential PPP2CA substrates and establish targeted degradation as a robust tool to unveil phosphatase substrates in cells.

## Highlights

• Generated ^*dTAG/dTAG*^*PPP2CA* homozygous knock-in HEK293 cells
• The PROTAC dTAG-13 selectively and efficiently degraded endogenous dTAG-PPP2CA
• Quantitative phospho-proteomics revealed 2204 phospho-proteins as putative PPP2CA substrates
• Putative PPP2CA substrates mapped to roles in spliceosome, RNA transport, and cell cycle

## Graphical abstract

**Figure:**
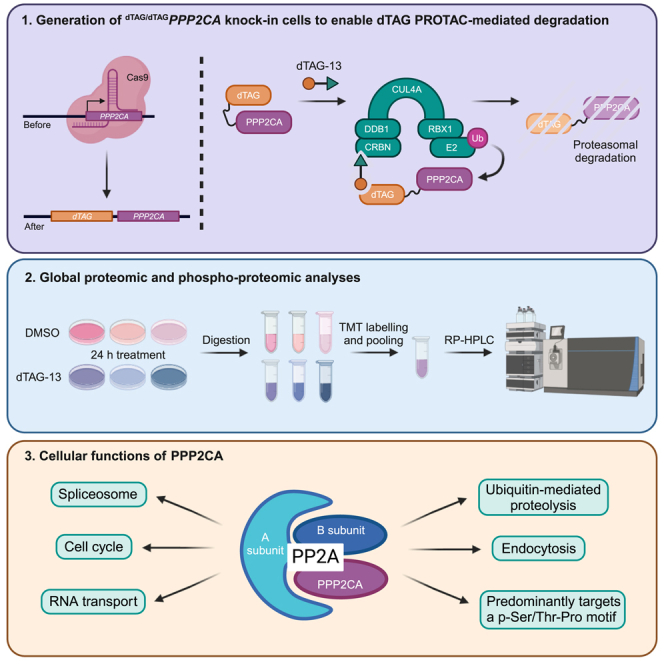

## Introduction

Protein phosphorylation is a fundamental post-translational modification that controls virtually every cellular process. Phosphorylation is a reversible event comprising the addition of a phosphoryl group through formation of a hydrolysable phospho-ester bond. In proteins, this modification occurs primarily on serine, threonine, or tyrosine residues. In response to specific signals, phosphorylation can alter protein conformation, stability, catalytic activity, subcellular localization, or interactions with other partners, thereby altering protein function, cell signaling and, ultimately, cellular fate decisions. Protein kinases catalyze the attachment of a phosphoryl group to target proteins while phosphatases elicit hydrolysis to remove the group. Balancing the activities of these enzymes enables fine-tuning of protein phosphorylation states and, consequently, these enzymes serve crucial roles in metabolism, maintaining homeostasis, cellular transport, and secretory processes. Unsurprisingly, numerous human diseases are associated with aberrant regulation of protein phosphorylation, including many cancers and neurodegenerative disorders.^1^^,^^2^^,^^3^ For example, hyper-activation of human epidermal growth factor receptor 2 (HER2) kinase, which leads to increased mitogen-activated protein kinase (MAPK) pathway signaling and proliferation, has been linked to breast cancer.^4^ Similarly, hyper-phosphorylation of tau protein by kinases such as cyclin-dependent kinase 5 (CDK5) and glycogen synthase kinase 3β (GSK3β) has been implicated in the formation of neurofibrillary tangles that have been associated with development of Alzheimer’s disease.^5^ As such, protein kinases and phosphatases have been explored as potential drug targets. While many specific kinase inhibitors have been developed, with around 75 approved for clinical use by the US Food and Drug Administration (FDA),^6^^,^^7^^,^^8^ equivalent progress has not been seen in the targeting of phosphatases. This is perceived to be a result of the lack of substrate specificity displayed by phosphatases.^9^ Nonetheless, the full substrate landscape for different phosphatases remains poorly defined. More than two-thirds of the 518 kinases in the human kinome conduct phosphorylation of serine and threonine residues, attributing to around 98% of documented phosphorylation events and forming one of the most common cellular post-translational modifications.^10^ Yet the majority of serine and threonine dephosphorylation is performed by only two phosphatases: protein phosphatase 1 (PP1) and protein phosphatase 2A (PP2A).^11^

PP2A is an essential Ser/Thr phosphatase that belongs to the PPP (phospho-protein phosphatase) family. PP2A has been implicated in a broad range of cellular processes such as proliferation, DNA repair, RNA splicing, and apoptosis, and is widely considered to display tumor suppressor functions.^12^ Indeed, activation of PP2A through PP2A-activating drugs (PADs) is being investigated as a potential novel drug mechanism to tackle some cancers as well as neurodegenerative and inflammation-mediated diseases.^13^ Although PP2A is reported to exist in a dimeric complex, comprising a scaffolding A subunit and catalytic C subunit, there is overwhelming evidence that it exists as a trimeric holoenzyme complex where a regulatory B subunit is also involved (Figure S1).^14^^,^^15^^,^^16^^,^^17^ The trimeric holoenzyme configuration functions to regulate PP2A phosphatase activity with the B subunit providing temporal and spatial selectivity.^18^^,^^19^ The regulatory B subunits are encoded by 15 different genes and have at least 26 different transcript and splice variants, which are classified into four families: B/B55, B’/B56, B’’/PR72, and B‴/Striatin.^12^ The B subunits provide substrate selectivity for PP2A through assembly of over 70 possible holoenzyme complexes.^20^ In contrast to the B subunits, there are only two isoforms each of the scaffolding A subunit (PR65A and B) and the catalytic C subunit (PPP2CA and B). The PPP2CA and PPP2CB isoforms of the catalytic subunit share 97% homology, with variation occurring at the N-terminus.^21^ Nonetheless, PPP2CA is typically over 10-fold more abundant than PPP2CB in most cells due to stronger promoter activity and differences in messenger RNA (mRNA) turnover rates.^22^ Interestingly, null mutation of *PPP2CA* results in early embryonic lethality, indicating that, despite high sequence similarity, PPP2CB is unable to sufficiently compensate for the loss of PPP2CA and associated catalytic activity.^23^ These findings suggest that reducing PP2A catalytic activity to below a certain threshold is not tolerated and that non-redundant functions may exist for PPP2CA and PPP2CB, at least in the context of embryonic development.

In addition to being regulated by the formation of the holoenzyme complex, PPP2CA catalytic activity is also tightly controlled through post-translational modification of the C-terminus by phosphorylation and methylation, which serve to promote or impede interaction with regulatory B subunits.^20^ Furthermore, the presence of similar short sequences, known as short linear interaction motifs (SLiMs), within PP2A substrates has been identified to promote interaction with specific regulatory B subunits for their dephosphorylation by the holoenzyme complex. The SLiM motif LxxIxE, particularly LSPIxE, has been implicated in binding B56α subunits,^24^^,^^25^^,^^26^ while p[ST]-P-x(4,10)-[RK]-V-x-x-[VI]-R, found in PP2A substrates such as tau, was associated with B55α engagement.^27^

To explore the extent of PP2A holoenzyme activity and define properties of putative PP2A substrates, some phospho-proteomic studies have been reported. One such study identified phospho-proteins from calyculin A-treated HeLa cell extracts that were lost upon incubation of the extracts with recombinant PPP2CA *in vitro.*^28^ From this study, it was suggested that PPP2CA intrinsically favors dephosphorylation of phospho-Thr residues. Another study employed an *in vitro* assay system called MRBLE:Dephos to ascertain PP2A-B55 and PP1 amino acid preferences before uncovering putative PP2A-B55 substrates in mitotic exit using phospho-proteomics.^29^ Phospho-proteomic analysis upon short interfering RNA (siRNA)-mediated depletion of PP2A inhibitors, such as cancerous inhibitor of protein phosphatase 2A (CIP2A), SET nuclear proto-oncogene (SET) and protein phosphatase methylesterase 1 (PME-1), or the A scaffolding subunit PPP2R1A, resulted in identification of putative PP2A substrates predicted to be involved in many cellular processes, including RNA splicing, kinase signaling, and DNA repair.^30^ Other studies have explored PP2A substrates controlled by specific holoenzyme configurations or B subunit isoforms (such as PPP2R2A/B55α,^31^ PP2A-B55/B, -B56/B′ and -PR48/B’’,^32^ PP2A-B55/B and PP2A-B56/B’,^26^ PPP2CB^33^ and PP2A-Cdc55 in *Saccharomyces cerevisiae*^34^), or by probing PP2A activity in response to specific stimuli, such as downstream of modulation of PP2A inhibitors/activators.^35^ An approach involving disruption of the core PPP2CA catalytic subunit *in cellulo*, followed by an unbiased phospho-proteomic analysis, could enable delineation of the full range of PPP2CA substrates within that cellular context. However, due to the essential function of PPP2CA for cell survival, its sustained depletion, for example with clustered regularly interspaced short palindromic repeats (CRISPR)/Cas9 genome editing, is not feasible.

Inducible protein degradation can overcome the limitations of prolonged disruption of essential genes and has been employed to interrogate phosphatase function, for example through auxin-mediated degradation of the catalytic subunit of Ser/Thr phosphatase PP6 (PP6c).^36^ Recent advances in the targeted protein degradation field have enabled the efficient and selective acute degradation of proteins of interest (POIs) through small heterobifunctional molecules, known as proteolysis-targeting chimeras (PROTACs) ^37^^,^^38^ PROTACs harness endogenous cellular degradation machinery, such as the ubiquitin proteasome system, thus avoiding the exogenous TIR1 (transport inhibitor response 1) expression that is required for the auxin-inducible degradation system. By tagging POIs endogenously with degron tags, such as the dTAG (FKBP12^F36V^), using CRISPR/Cas9 genome editing, PROTACs directed at the dTAG, such as dTAG-13,^39^ dTAG^V^-1,^40^ and dTAG-VHL,^41^ can lead to the degradation of POIs. Here, we employ dTAG-PROTAC technology to target the degradation of dTAG-PPP2CA, which was knocked-in homozygously to human embryonic kidney (HEK) 293 cells using CRISPR/Cas9 genome editing, before employing unbiased phospho-proteomic analysis to reveal the full repertoire of putative PPP2CA substrates in this cellular context.

## Results

### Generation of ^dTAG/dTAG^*PPP2CA* knock-in HEK293 cells and assessment of PROTAC-mediated degradation

**Figure 1:**
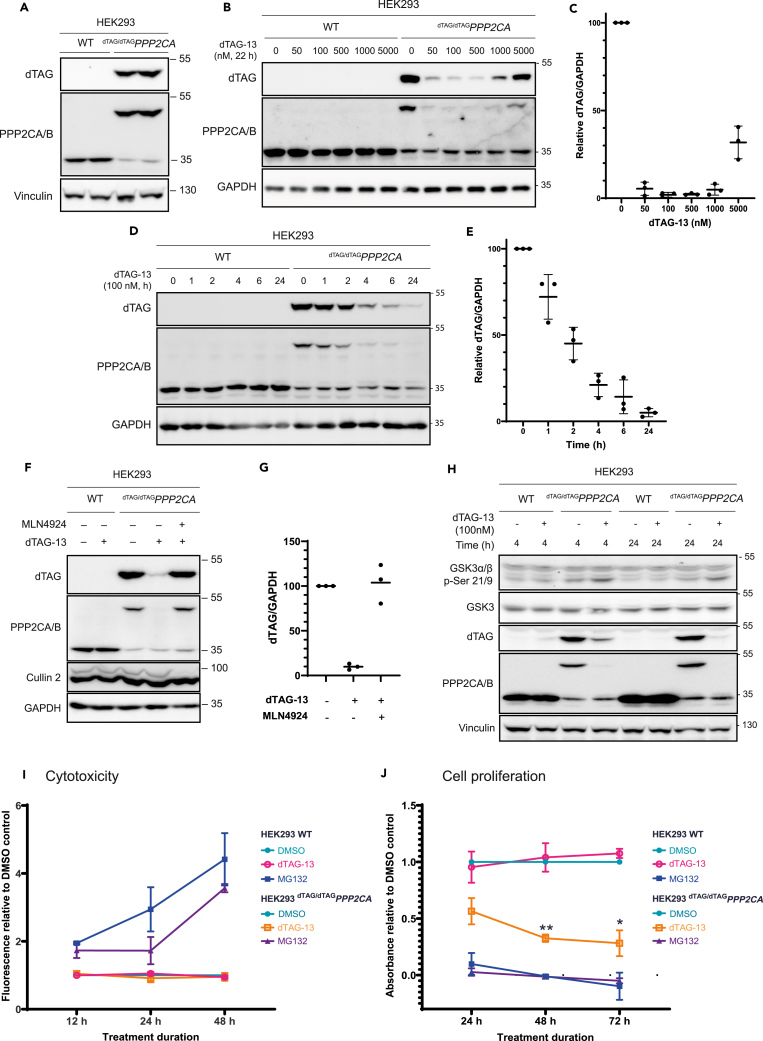
Degradation of dTAG-PPP2CA with dTAG-13 (A) Characterization of ^dTAG/dTAG^*PPP2CA* HEK293 knock-in cell lines. Extracts (20 μg protein) from wild type (WT) and ^dTAG/dTAG^*PPP2CA* HEK293 cells were resolved by SDS-PAGE and transferred to nitrocellulose membranes, which were analyzed by immunoblotting with the indicated antibodies. Anti-FKBP12 and anti-dTAG antibodies were used to detect dTAG, also known as FKBP12^F36V^, with anti-FKBP12 antibody raised against a WT FKBP12 antigen and anti-dTAG raised against a FKBP12^F36V^ antigen, but both capable of detecting dTAG/FKBP12^F36V^. (B and C) Dose response of dTAG-13-mediated dTAG-PPP2CA degradation. As in (A), except extracts (20 μg protein) were from WT and ^dTAG/dTAG^*PPP2CA* HEK293 cells treated for 22 h with the indicated concentrations of dTAG-13 or DMSO. Relative dTAG-PPP2CA levels from immunoblots (B) were quantified and are presented in (C) as mean values ±SD from n = 3 independent experiments. Anti-FKBP12 antibody was used to detect dTAG (FKBP12^F36V^). (D and E) Time course of dTAG-13-mediated dTAG-PPP2CA degradation. As in (B and C), except extracts (20 μg protein) were from WT and ^dTAG/dTAG^*PPP2CA* HEK293 cells treated with 100 nM dTAG-13 for the indicated time durations. (F and G) Degradation of dTAG-PPP2CA in ^dTAG/dTAG^*PPP2CA* HEK293 cells using optimized conditions. As in (B and C), except extracts (20 μg protein) were from ^dTAG/dTAG^*PPP2CA* HEK293 cells treated with DMSO, dTAG-13 (100 nM) or a combination of dTAG-13 (100 nM) and MLN4924 (1000 nM) for 24 h prior to lysis. (H) Confirmation that dTAG-PPP2CA retains phosphatase activity following dTAG knock-in. As in (A), except extracts (20 μg protein) were from WT and ^dTAG/dTAG^*PPP2CA* HEK293 cells treated with DMSO or dTAG-13 (100 nM) for 4 h or 24 h prior to lysis. (I) Cell cytotoxicity assay to determine cytotoxicity of dTAG-13-mediated dTAG-PPP2CA degradation in ^dTAG/dTAG^*PPP2CA* and WT HEK293 cells. Cells were treated for 12, 24 or 48 h with dTAG-13 (100 nM), DMSO as a negative control or MG132 (40 μM) as a positive control. Cytotoxicity was assessed by using the CellTox Green Assay (Promega), with fluorescence measured using a PHERAstar plate reader (ex: 480 nm em: 530 nm). Data represent n = 3, with 3 technical replicates included per condition for each separate biological repeat. Values are shown as a mean fluorescence reading normalized to DMSO controls ±SD. (J) Cell proliferation assay to determine impact of dTAG-13-mediated dTAG-PPP2CA degradation on cell proliferation. Proliferation was measured using the CellTiter 96 AQ_ueous_ One Solution Cell Proliferation Assay (Promega) by treating ^dTAG/dTAG^*PPP2CA* and WT HEK293 cells with dTAG-13 (100 nM) for 24, 48 or 72 h, with DMSO as a negative control or MG132 (40 μM) as a positive control. Cells were then incubated with the CellTiter 96 AQ_ueous_ One Solution Reagent before absorbance was measured at 490 nm. Data represent n = 3, with 3 technical replicates included per condition for each separate biological repeat. Values are shown as a mean absorbance reading normalized to DMSO controls ±SD, with two-way ANOVA and Tukey’s HSD post-hoc test used for statistical analysis.

To explore the substrates of PPP2CA, we generated ^dTAG/dTAG^*PPP2CA* knock-in HEK293 cells in which dTAG was homozygously inserted at the N-terminus of *PPP2CA* by using CRISPR/Cas9 technology^42^ (Figures S2A–S2F). Successful knock-in of dTAG was confirmed by a combination of immunoblotting with anti-PPP2CA/B and anti-dTAG antibodies (Figures 1A and S2C) as well as polymerase chain reaction (PCR) amplification and genomic sequencing of the target gene locus (Figures S2D–S2F). A cross-reacting band at ∼48 kDa, the expected molecular weight for dTAG-PPP2CA, was detected by both anti-dTAG and anti-PPP2CA/B immunoblotting of extracts from the ^dTAG/dTAG^*PPP2CA* knock-in HEK293 cell clone, which was absent in wild type (WT) HEK293 cells, confirming the knock-in (Figures 1A and S2C). The band at ∼35 kDa observed with anti-PPP2CA/B antibody represents the endogenous PPP2CA and PPP2CB, since the two isoforms share 100% sequence identity for the epitope that the antibody was raised against (residues 289–307 of human PPP2CA). Consistent with this, a reduction in the intensity of this 35 kDa band was observed with anti-PPP2CA/B immunoblotting in ^dTAG/dTAG^*PPP2CA* HEK293 cells compared to WT HEK293 cells (Figures 1A and S2C). To ensure homozygous knock-in, the genomic locus around ^dTAG/dTAG^*PPP2CA* from clone 1 knock-in cells was amplified by PCR and yielded one product at the expected ∼2800 bp molecular weight, compared to ∼1200 bp as was observed in WT cells (Figure S2D). The PCR product from ^dTAG/dTAG^*PPP2CA* cells was sequenced and confirmed homozygous knock-in at the expected locus (Figures S2E and S2F).

Next, we sought to explore the targeted degradation of dTAG-PPP2CA with dTAG-13.^39^ WT and ^dTAG/dTAG^*PPP2CA* HEK293 cells were treated with increasing concentrations of dTAG-13 (from 50 to 5000 nM) or dimethylsulphoxide (DMSO) for 22 h. In comparison to DMSO controls, a dose-dependent degradation of dTAG-PPP2CA was observed from 50 to 500 nM dTAG-13 in ^dTAG/dTAG^*PPP2CA* HEK293 cells with almost complete degradation observed with dTAG-13 treatment at 100 and 500 nM (Figures 1B and 1C). At 1000 and 5000 nM dTAG-13, the degradation of dTAG-PPP2CA was less efficient (Figure 1B), implying a hook effect, which is reminiscent of PROTACs at high treatment concentrations.^43^ Importantly, dTAG-13 treatment, even at the highest concentration of 5000 nM, did not elicit any change in the abundance of endogenous PPP2CA in WT HEK293 cells (Figure 1B). A time course experiment in which WT and ^dTAG/dTAG^*PPP2CA* HEK293 cells were treated with 100 nM dTAG-13 showed a time-dependent degradation of dTAG-PPP2CA, with ∼50% degradation observed at 2 h and almost complete degradation observed at 24 h (Figures 1D and 1E). Again, no change in the abundance of endogenous PPP2CA was observed in WT HEK293 cells at any time point following dTAG-13 treatment (Figure 1D). In comparison to other dTAG-targeting PROTACs, including dTAG^V^-1^40^ and dTAG-VHL,^41^ dTAG-13 achieved optimal degradation of dTAG-PPP2CA with the lowest PROTAC concentration, so we continued with dTAG-13 (Figures S3A–S3D). dTAG-13 recruits dTAG to the cullin 4A (CUL4A)-ring-box 1 (RBX1) E3 ligase complex via the CUL4A substrate receptor cereblon (CRBN) for degradation by the proteasome.^39^ Consistent with this, treatment of ^dTAG/dTAG^*PPP2CA* HEK293 cells with the NEDD8 activating enzyme E1 subunit 1 (NAE1) inhibitor MLN4924, which prevents the NEDDylation and activation of all cullins,^44^ resulted in the rescue of dTAG-PPP2CA degradation caused by dTAG-13 (Figures 1F and 1G). Compared to DMSO treatment, MLN4294 treatment led to a collapse of the upper cullin 2 (CUL2) band corresponding to NEDDylated species, confirming inhibition of NAE1 (Figures 1F and 1G).

We also set out to test whether the incorporation of dTAG had any impact on the ability of PPP2CA to form a holoenzyme complex with other PP2A subunits or on the catalytic activity of the resulting holoenzyme. Anti-HA immunoprecipitation of extracts from HEK293 cells over-expressing HA-PPP2CA or HA-dTAG-PPP2CA confirmed that HA-dTAG-PPP2CA maintained interaction with A and B subunits, indicating that holoenzyme complex formation was not interrupted by the incorporation of dTAG on PPP2CA (Figure S2G). This is consistent with previous work where we demonstrated that PPP2CA tagged with a FLAG tag and an anti-GFP nanobody (FLAG-aGFP_6M_-PPP2CA) still interacts with other PP2A holoenzyme subunits.^45^ We next explored whether endogenously expressed dTAG-PPP2CA retained phosphatase catalytic activity against a reported PP2A substrate, namely *p*-Ser9 GSK3β.^46^^,^^47^^,^^48^^,^^49^ In DMSO-treated WT and ^dTAG/dTAG^*PPP2CA* HEK293 cells over 4 and 24 h duration, no substantial change in the abundance of *p*-Ser9 GSK3β was observed between the cell lines, suggesting that the dTAG knock-in on *PPP2CA* had no overt effect on the basal dephosphorylation of *p*-Ser9 GSK3β (Figure 1H). The degradation of dTAG-PPP2CA with dTAG-13 in ^dTAG/dTAG^*PPP2CA* HEK293 cells would be predicted to enhance the abundance of *p*-Ser9 GSK3β. Indeed, dTAG-13 treatment of ^dTAG/dTAG^*PPP2CA* HEK293 cells for both 4 h and 24 h caused a marked degradation of dTAG-PPP2CA and concurrently led to enhanced levels of *p*-Ser9 GSK3β compared to DMSO-treated controls (Figure 1H). In WT HEK293 cells, no differences in *p*-Ser9 GSK3β levels were apparent with either DMSO or dTAG-13 treatment at both durations (Figure 1H). GSK3β protein abundance was unaffected by the ^dTAG/dTAG^*PPP2CA* knock-in or the treatments with either DMSO or dTAG-13.

We then explored whether targeted degradation of dTAG-PPP2CA impacted cell viability and proliferation. We observed no significant cell toxicity up to 48 h following dTAG-13 treatment, in either WT or ^dTAG/dTAG^*PPP2CA* HEK293 cells (Figure 1I). However, prolonged degradation of dTAG-PPP2CA with dTAG-13 treatment for 48 h and 72 h significantly impeded cell proliferation (Figure 1J), consistent with previous reports of the importance of PPP2CA to cell proliferation.^12^^,^^23^^,^^50^^,^^51^ Collectively, these data reveal optimal conditions for the dTAG-13-mediated degradation of dTAG-PPP2CA in ^dTAG/dTAG^*PPP2CA* HEK293 cells and support that this inducible, targeted degradation could enable dissection of the plethora of substrates whose phospho-regulation is controlled by PPP2CA.

### A global total- and phospho-proteomic analysis to elucidate PPP2CA targets

**Figure 2:**
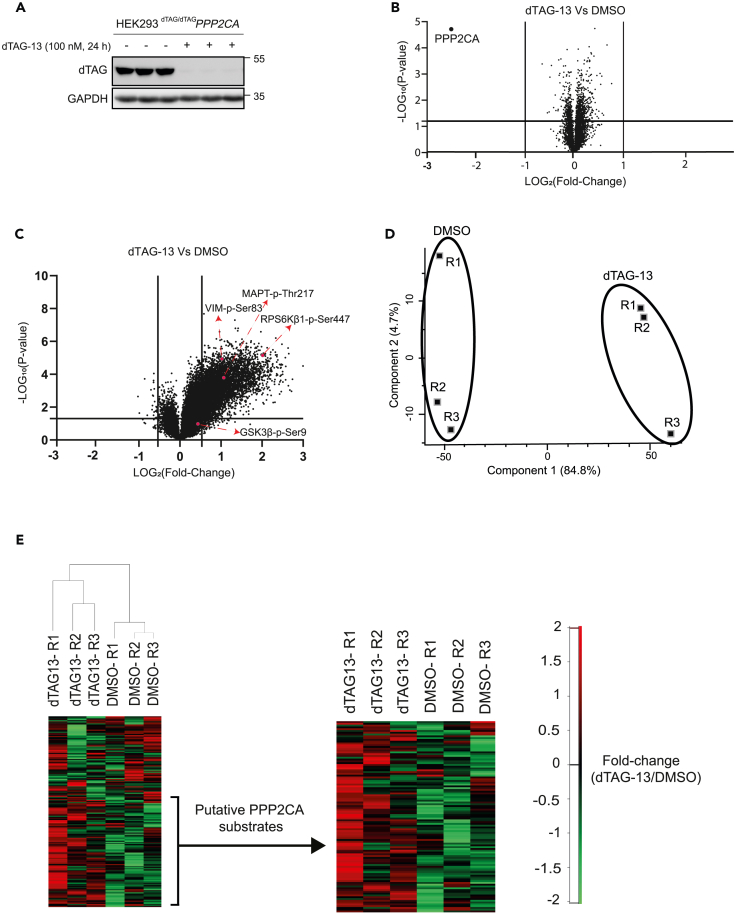
Global proteomic and phospho-proteomic analysis upon PPP2CA degradation (A) Extracts (20 μg protein) from ^dTAG/dTAG^*PPP2CA* HEK293 cells treated for 24 h with DMSO or dTAG-13 (100 nM) (3 independent treatments per condition) prior to lysis were resolved by SDS-PAGE and transferred to nitrocellulose membranes, which were analyzed by immunoblotting with the indicated antibodies. (B) Extract samples from (A) were analyzed using the TMT-based proteomics workflow, as described in Figure S4. Volcano plot showing quantitative changes in the identified proteins. Data plotted represent log_2_ of the fold change in protein abundance in dTAG-13-treated extracts normalized to DMSO-treated controls against −log_10_ of the p value for each identified protein. Under these conditions, the only protein that was significantly degraded (fold change < 0.5) upon dTAG-13 treatment was dTAG-PPP2CA. (C) Volcano plot showing global phospho-proteome alteration in dTAG-13-treated ^dTAG/dTAG^*PPP2CA* HEK293 cells compared to DMSO-treated controls. Data plotted represent log_2_ of the fold change of phospho-peptides identified in dTAG-13-treated extracts normalized to DMSO-treated controls against −log_10_ of the p value for each phospho-peptide. The positions of some phospho-proteins of interest are indicated by arrows. (D) Principal component analysis of global quantitative phospho-proteomics data across the three biological replicates (R1-3) for each condition. (E) Unsupervised clustering of altered phospho-peptides identified in dTAG-13- and DMSO-treated ^dTAG/dTAG^*PPP2CA* HEK293 cells. ANOVA was used to identify significantly altered phospho-peptides upon dTAG-PPP2CA degradation. Abundance values of differentially phosphorylated peptides are represented in a heatmap format, with green representing low abundance and red representing high abundance. The sliding-scale for relative phospho-protein abundance is included. Phospho-peptides whose abundance significantly increased upon dTAG-13-mediated dTAG-PPP2CA degradation, indicating them as putative PPP2CA substrates, are shown with increased magnification.

To uncover PPP2CA targets in ^dTAG/dTAG^*PPP2CA* HEK293 cells, unbiased tandem mass tag (TMT)-based quantitative proteomic and phospho-proteomic workflows were adopted following degradation of dTAG-PPP2CA with 100 nM dTAG-13 treatment for 24 h (Figure S4). DMSO-treated cells were included as a negative control. Immunoblot analysis of extracts from 3 biological replicates, which were prepared for proteomic and phospho-proteomic analysis, demonstrated a robust degradation of dTAG-PPP2CA in ^dTAG/dTAG^*PPP2CA* HEK293 cells upon treatment with dTAG-13 for 24 h in comparison to DMSO-treated controls (Figure 2A). Total quantitative proteomic analysis of DMSO- and dTAG-13-treated ^dTAG/dTAG^*PPP2CA* HEK293 cell extracts identified a total of 80,998 peptides, belonging to 7,589 unique proteins (full list available in Table S1). Of these, the only protein whose abundance was significantly reduced (p < 0.05 and fold change greater than 2-fold) in dTAG-13-treated cells compared to DMSO-treated controls was PPP2CA (Figure 2B), demonstrating the remarkable selectivity of targeted degradation caused by dTAG-13. The quantitative total proteomic data demonstrating that PPP2CA was the only protein targeted for degradation by dTAG-13 prompted us to confidently proceed with the quantitative phospho-proteomic approach to determine putative PPP2CA substrates. Quantitative phospho-proteomic analysis in DMSO- and dTAG-13-treated ^dTAG/dTAG^*PPP2CA* HEK293 cell extracts identified a total of 39,103 phospho-peptides belonging to 5,829 proteins (full list available in Table S2). Of these, 2,651 phospho-peptides corresponding to 1,149 proteins showed a significant increase in abundance of >2-fold in dTAG-13-treated cells compared to DMSO-treated controls, while 6,280 phospho-peptides corresponding to 2,204 proteins showed a significant increase in abundance of >1.5-fold (Figure 2C). These phospho-peptides denote potential PPP2CA substrates in these cells under the experimental conditions employed. In contrast, only 16 phospho-peptides belonging to 11 proteins were found to be significantly reduced in abundance (fold change greater than 1.5-fold) in dTAG-13-treated cells compared to DMSO-treated controls, which could imply indirect phosphorylation of targets that is dependent on PPP2CA activity or abundance.

To ensure concordance in identified phospho-peptides between the biological replicates for both DMSO- and dTAG-13-treated cells, a principal component analysis (PCA) was conducted using Perseus (Figure 2D). Indeed, PCA revealed that individual replicates from each group cluster together, with a clear separation between DMSO and dTAG-13 groups (Figure 2D). A heatmap was generated through hierarchical clustering of the identified phospho-peptides and potential PPP2CA substrates, which denote phospho-peptide enrichment in dTAG-13-treated replicates relative to DMSO-treated replicates, are indicated (Figure 2E). We took a selection of some interesting identified phospho-peptides and analyzed the significance in their phosphorylation upon dTAG-PPP2CA degradation using violin plots (Figure S5). These data clearly demonstrate a significant enrichment of these phospho-peptides upon dTAG-PPP2CA degradation, suggesting them to be putative PPP2CA substrates.

### *In silico* analysis of phospho-peptides and proteins identified as potential PPP2CA targets

**Figure 3:**
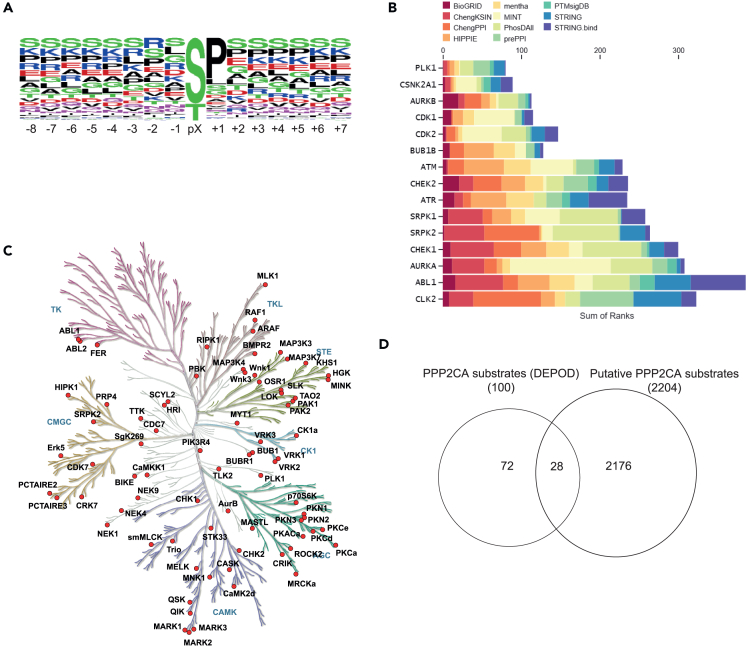
Deconvolution of the significantly enriched phospho-peptides upon dTAG-PPP2CA degradation (A) Motif analysis from the significantly enriched phospho-peptides upon dTAG-PPP2CA degradation. (B) Bar chart representing the MeanRank visualization from the kinase enrichment analysis based on phospho-proteins enriched upon dTAG-PPP2CA degradation. The top 15 kinases are plotted against the integrated ranking of the predicted kinases known to phosphorylate the identified phospho-proteins across different libraries, based on MeanRank score. The bar is colour-coded to reflect each library used for data analysis. (C) Protein kinases for which phospho-peptides were significantly enriched upon dTAG-PPP2CA degradation are indicated on the kinome map, which was generated using the KinMap tool. These kinases are potentially regulated by PPP2CA. (D) Venn diagram comparison between the identified putative PPP2CA substrates from our datasets and the reported PP2A substrates from the DEPOD database.

To elucidate whether the phospho-peptides enriched upon dTAG-PPP2CA degradation conform to a consensus dephosphorylation motif, a multiple sequence alignment was conducted for phospho-peptides that were enriched >1.5-fold in dTAG-13-treated cells over DMSO-treated controls, by using 16-mer peptides with the identified phospho-Ser/Thr residue placed in the middle.^52^^,^^53^ Consistent with the reported role of PPP2CA in dephosphorylating *p*-Ser/p-Thr residues, all enriched phospho-events were observed on Ser and Thr residues, with a majority (70%) observed on Ser residues (Figure 3A). The contrast in this observation to previous research may be owed to the normalization for the rate of natural occurrence of amino acids that was conducted in that research, or to the specific cellular context explored in our study. In almost 50% of the identified phospho-peptides, enrichment was observed for peptides possessing a Pro residue at the +1 position. This is consistent with previous studies that have reported some PP2A-mediated dephosphorylation of *p*-Ser/Thr residues that are phosphorylated by Pro-directed kinases, such as sites on microtubule-associated protein tau.^54^^,^^55^ At the −1 position, enrichment of Ser, Leu, Gly, Arg, Asp, and Lys residues was observed, indicating a tolerance for charged or hydrophobic residues immediately N-terminus of the phospho-site. Further upstream and downstream of the phospho-site, enrichment was seen for Ser, Lys, Arg, Pro, Glu, and Thr residues, suggesting that PPP2CA dephosphorylation weakly favors upstream and downstream residues that possess electrically charged or polar side chains. Enrichment of Ser and Thr residues both upstream and downstream of the phospho-site may imply that PPP2CA dephosphorylates clusters of multiple phospho-Ser/Thr residues. Indeed, among the phospho-peptides enriched upon dTAG-PPP2CA degradation, ∼4000 were identified as mono-phosphorylated, ∼1500 as di-phosphorylated and ∼300 as tri-phosphorylated (Figure S6A). Having explored the consensus motif of the PPP2CA-regulated phospho-sites, we were interested to explore the prevalence of known PP2A-B56α SLiM motif LSPIxE in our identified putative PPP2CA substrates. For this, FIMO (Find individual Motif Occurrences) was employed to scan the human proteome for the motif LSPIxE, which identified 897 unique proteins as containing this SLiM. Of these, 99 were identified by our phospho-proteomic analysis (Figure S6B), suggesting these may be PP2A-B56α substrates. Absence of this SLiM from other identified putative PPP2CA substrates potentially indicates other modes of substrate recruitment. In a similar vein, failure to identify other LSPIxE motif-containing proteins as potential PPP2CA substrates in our study could imply either that these proteins are not substrates of PPP2CA or that an appropriate biological context was not present to enable their identification.

We undertook an *in silico* analysis to identify the upstream kinases whose substrates are overrepresented in our identified putative PPP2CA substrate phospho-proteins. Through the Kinase Enrichment Analysis 3 tool, we found Ser/Thr-protein kinase PLK1, casein kinase 2α (CK2α), aurora kinase A and B (AURKA/B), cyclin-dependent kinase 1 and 2 (CDK1 and CDK2), BUB1B/BUBR1, ataxia telangiectasia mutated (ATM), checkpoint kinase 1 and 2 (CHEK1/2), ataxia telangiectasia and Rad3-related protein (ATR), Ser/Arg-rich splicing factor protein kinase 1 and 2 (SRPK1/2), Tyr protein kinase ABL1 and dual specificity CDC-like kinase 2 (CLK2) as among the top kinases known to regulate the phospho-proteins that we identified as putative PPP2CA substrates (Figure 3B). We also mapped all protein kinases within the human kinome tree using KinMap and reveal 50 kinases whose phosphorylation at certain residues was enriched upon dTAG-PPP2CA degradation (Figure 3C), suggesting PPP2CA activity might regulate the activity or function of these kinases, which are involved in cell processes such as mitosis, proliferation, and gene expression.^56^^,^^57^^,^^58^ Additionally, we compared the identified putative PPP2CA substrates against the reported PPP2CA substrates on the Human Dephosphorylation Database, DEPOD.^59^ Of the 2,204 most significantly enriched phospho-proteins identified, only 28 were reported by the DEPOD as PP2A substrates (Figure 3D), indicating that our study has provided a step change in the number of phospho-proteins reported as potential PPP2CA substrates. Furthermore, comparison with previous studies found the putative PPP2CA substrates identified in our study matched 262 out of 515 phospho-proteins identified as *in vitro* PPP2CA substrates^28^ (Figure S6C) and 345 out of 522 phospho-proteins determined to be PPP2R1A substrates^30^ (Figure S6D). In each case, our approach identified >1800 unique phospho-proteins as putative PPP2CA substrates. Encouragingly, when comparing to the DEPOD database, Enrichr listed PPP2CA as the phosphatase predicted to be the most likely to dephosphorylate the phospho-proteins we identified in our study as putative PPP2CA substrates (Figure S6E).

### *In silico* prediction of biological roles of the phospho-proteins identified as putative PPP2CA substrates

**Figure 4:**
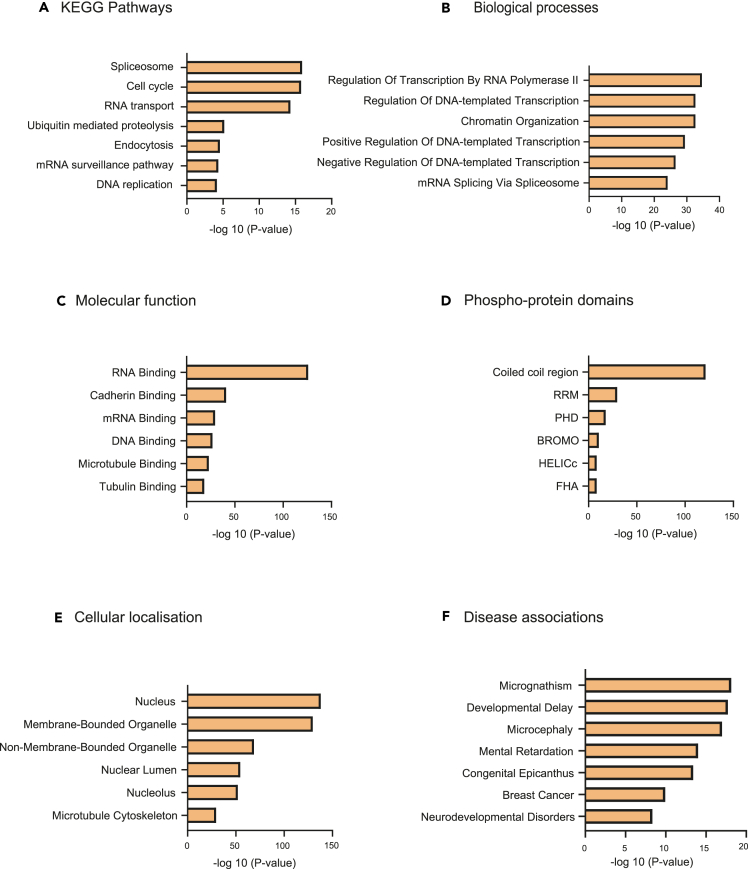
Gene Ontology (GO) analysis of proteins identified as putative PPP2CA substrates (A) Involvement of the identified putative PPP2CA substrates in KEGG (Kyoto Encyclopedia of Genes and Genomes) pathways. (B) Involvement of the identified putative PPP2CA substrates in different biological processes. (C) Known and predicted molecular functions of the identified putative PPP2CA substrates. (D) Domain architecture of proteins identified as putative PPP2CA substrates. (E) Known subcellular distribution of the identified putative PPP2CA substrates. (F) Disease associations of the identified putative PPP2CA substrates. For A-F, the data are represented as bar charts indicating the p value for associations of the identified putative PPP2CA substrates to given biological pathways (A), cellular processes (B), molecular functions (C), domain architectures (D), subcellular distribution (E) and diseases (F). These values were generated using gene ontology (GO) analysis and a selection of the top classes has been included in the bar charts.

For the identified phospho-proteins that were enriched upon dTAG-PPP2CA degradation, we exploited *in silico* analysis to explore their reported involvement in cell signaling pathways, biological processes, molecular function, protein domain architecture, subcellular distribution, disease associations, and protein-protein interactions (Figures 4A–4F and S7). Analyses were conducted using the Database for Annotation, Visualization and Integrated Discovery (DAVID), Enrichr, FunRich, and STRING^60^^,^^61^^,^^62^^,^^63^^,^^64^^,^^65^^,^^66^ and the full lists of results can be found in Table S3. Kyoto Encyclopedia of Genes and Genomes (KEGG) pathway analysis revealed that a significantly high percentage of the identified phospho-proteins are known to be involved in spliceosome function, the cell cycle, RNA transport and surveillance, ubiquitin-mediated proteolysis, endocytosis, and DNA replication (Figure 4A). The identified phospho-proteins were also significantly implicated in biological processes including regulation of transcription by RNA polymerase II, DNA transcription, chromatin organization, and mRNA splicing (Figure 4B). These observations were supported by the reported molecular functions of the identified phospho-proteins, which showed a significantly high percentage to be involved in binding RNA, cadherin, mRNA, DNA, microtubules, and tubulin (Figure 4C). When we performed a network analysis of the phospho-proteins, major functional groups identified showed interaction of groups involved in the spliceosome, RNA transport, DNA replication, DNA repair, and the cell cycle (Figure S7), supporting the aforementioned KEGG pathway, biological processes and molecular function associations. Domain architecture analysis of the identified phospho-proteins revealed that 43% contained coiled-coil domains, which are prominent in transcriptional regulation (Figure 4D). Other domains that showed a significant presence in the identified phospho-proteins included the RNA recognition motif (RRM) (5.6%), plant homeodomain (PHD) (2.8%), bromo domain (BROMO) (1.5%), helicase superfamily c-terminal domain (HELICc) (2.4%), and the forkhead-associated (FHA) domain (FHA) (1.1%). Analysis of the subcellular distribution of the identified phospho-proteins indicated presence in many subcellular compartments, including the nucleus, membrane- and non-membrane-bound organelles, and microtubule cytoskeleton (Figure 4E). Disease associations of the identified putative PPP2CA substrates showed involvement in micrognathism, developmental delay, microcephaly, mental retardation, congenital epicanthus, breast cancer, and neurodevelopmental disorders (Figure 4F).

### Validation of PPP2CA phospho-proteomic data by immunoblotting

**Figure 5:**
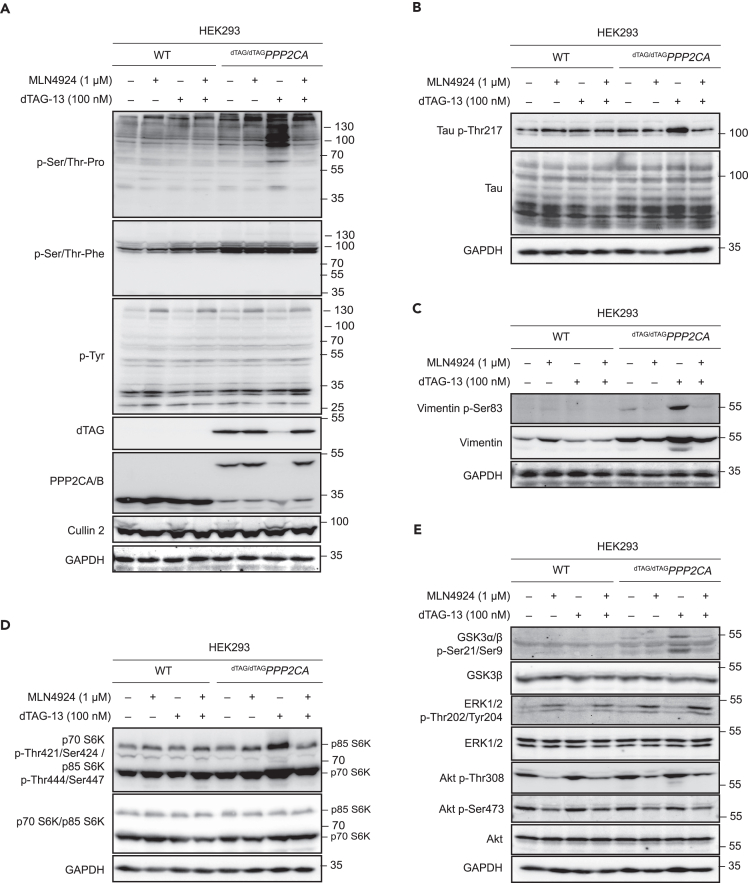
Immunoblotting validation of putative PPP2CA substrates (A–E) Extracts (20 μg protein) from WT and ^dTAG/dTAG^*PPP2CA* HEK293 cells treated with DMSO, dTAG-13 (100 nM), MLN4924 (1 μM) or dTAG-13 and MLN4924 for 24 h prior to lysis were resolved by SDS-PAGE and transferred to nitrocellulose membranes, which were analyzed by immunoblotting with the indicated antibodies. Data are representative of three independent experiments.

To validate some of the phospho-proteins identified by phospho-proteomics as potential PPP2CA targets, WT and ^dTAG/dTAG^*PPP2CA* HEK293 cells were treated with DMSO, MLN4924 (1 μM), dTAG-13 (100 nM) or dTAG-13 and MLN4924 for 24 h prior to lysis. Immunoblotting with anti-dTAG antibody confirmed the degradation of dTAG-PPP2CA in ^dTAG/dTAG^*PPP2CA* HEK293 cells treated with dTAG-13 in comparison to DMSO (Figure 5A). Treatment of ^dTAG/dTAG^*PPP2CA* HEK293 cells with MLN4924 alone had no effect on dTAG-PPP2CA abundance, while co-treatment of cells with MLN4924 and dTAG-13 rescued the dTAG-PPP2CA degradation caused by dTAG-13 (Figure 5A). Again, MLN4924 treatment resulted in inhibition of cullin NEDDylation and activation, evident by the band collapse of CUL2 in MLN4924-treated samples (Figure 5A). No changes in the abundance of endogenous PPP2CA/B in WT cells and PPP2CB in ^dTAG/dTAG^*PPP2CA* HEK293 cells were apparent when cells were treated with DMSO, MLN4294, dTAG-13, or dTAG-13+MLN4924 (Figure 5A). Under these conditions, we probed these extracts with phospho-specific antibodies against some of the phospho-proteins identified as potential PPP2CA targets from the phospho-proteomic analysis.

Phospho-peptides that conform to a phospho-Ser/Thr-Pro motif were observed to be overwhelmingly enriched upon dTAG-PPP2CA degradation by phospho-proteomic analysis (Figure 3A) and immunoblotting with an anti-phospho-Ser/Thr-Pro motif antibody corroborated these observations (Figure 5A). Levels of phospho-Ser/Thr-Pro were greatly enhanced upon degradation of dTAG-PPP2CA with dTAG-13 in ^dTAG/dTAG^*PPP2CA* HEK293 cells but not in WT HEK293 cells, in comparison to corresponding DMSO-treated controls (Figure 5A). MLN4924 treatment alone in both cells caused little difference to phospho-Ser/Thr-Pro signals relative to DMSO-treated controls. However, in ^dTAG/dTAG^*PPP2CA* HEK293 cells treated with dTAG-13, co-treatment with MLN4924, which prevented the degradation of dTAG-PPP2CA, caused a reduction in phospho-Ser/Thr-Pro signals to similar levels observed with DMSO or MLN4924 treatment alone. In contrast to the phospho-Ser/Thr-Pro motif, there was no discernible change in either phospho-Ser/Thr-Phe or phospho-Tyr levels across the samples (Figure 5A), which is consistent with the *in silico* analysis of the phospho-proteomic data that showed no enrichment of these motifs among the identified phospho-peptides (Figure 3A).

We also sought to validate individual potential PPP2CA substrates identified by phospho-proteomic analysis. Despite a limited availability of phospho-specific antibodies raised against the particular phospho-sites elucidated by phospho-proteomic analysis, we were able to validate a small number of these potential PPP2CA substrates. Firstly, our phospho-proteomic analysis identified a *p*-Thr534 peptide from tau to be significantly enriched by 1.9-fold in dTAG-13-treated ^dTAG/dTAG^*PPP2CA* HEK293 cells compared to DMSO-treated controls (Table S2). By immunoblotting with an anti-*p*-Thr217 (also known as *p*-Thr534) tau antibody, we observed a clear increase in *p*-Thr217 abundance in dTAG-13-treated ^dTAG/dTAG^*PPP2CA* HEK293 cells in comparison to DMSO-treated controls (Figure 5B). In contrast, treating WT HEK293 cells with dTAG-13 did not yield an increased *p*-Thr217 tau abundance in comparison to DMSO treatment (Figure 5B). The *p*-Thr217 signal was only detected on a tau species of ∼100 kDa, corresponding to Big-tau, which contains a large additional exon termed exon 4a.^67^ Total tau levels, regardless of the isoforms, remained similar across all treatment conditions. Co-treatment of ^dTAG/dTAG^*PPP2CA* HEK293 cells with dTAG-13 and MLN4924, which rescued dTAG-PPP2CA degradation, reduced the *p*-Thr217 tau abundance to levels seen with DMSO treatment or MLN4924 treatment alone (Figure 5B). PP2A has previously been reported to dephosphorylate tau at multiple residues, including Thr205, Thr212, Ser214, and Ser262.^54^ Of these, our phospho-proteomic screen significantly identified Thr212 and Ser214 from tau (Thr529 and Ser531) with fold changes of 1.9 and 1.2, respectively (Table S2).

Our phospho-proteomic analysis also revealed a significant 1.8-fold change of a phospho-peptide containing *p*-Ser83 of vimentin, which was previously reported to be hyper-phosphorylated upon PP2A inhibition (Table S2).^68^ By immunoblotting with anti-vimentin *p*-Ser83 antibody, we observed a substantial increase in *p*-Ser83-vimentin levels in dTAG-13-treated ^dTAG/dTAG^*PPP2CA* HEK293 cells in comparison to DMSO treatment, while a slight increase in total vimentin levels and an extra lower molecular weight species were also apparent (Figure 5C). Hyper-phosphorylation of vimentin is reported to cause disassembly of vimentin filaments into bundles around the nucleus,^68^ which may be related to the change in abundance and the appearance of an additional vimentin species that we observed here. No substantial changes in *p*-Ser83-vimentin levels were evident in WT HEK293 cells with any of the treatments.

A phospho-peptide containing *p*-Ser447 of p85 S6K was also identified in our phospho-proteomic analysis and experienced a significant 3.6-fold change in abundance in dTAG-13-treated ^dTAG/dTAG^*PPP2CA* HEK293 cells in comparison to DMSO-treated controls (Table S2). p85 S6K and p70 S6K are two protein isoforms formed through alternative splicing of the ribosomal protein S6 kinase β1 (RPS6Kβ1) gene, with p85 S6K containing an extra 23 amino acids at the N-terminus in comparison to the p70 S6K isoform.^69^ These S6K isoforms are Ser/Thr protein kinases that respond to mammalian target of rapamycin (mTOR) signaling and act downstream of phosphoinositide-dependent protein kinase 1 (PDPK1) to phosphorylate the S6 ribosomal protein, resulting in an increase in protein synthesis and cell proliferation.^70^^,^^71^ RPS6Kβ1 has been reported as a PP2A substrate previously.^72^^,^^73^^,^^74^ When immunoblotting with an anti-phospho-S6K antibody, which recognizes both *p*-Thr444/Ser447 p85 S6K and *p*-Thr421/Ser424 p70 S6K, a clear increase in signal intensity was observed for p85 S6K as well as p70 S6K in dTAG-13-treated ^dTAG/dTAG^*PPP2CA* HEK293 cells in comparison to DMSO- and dTAG-13-treated WT HEK293 cells (Figure 5D). Interestingly, the levels of phospho-S6K were seen to increase with MLN4924 treatment, in both WT and ^dTAG/dTAG^*PPP2CA* HEK293 cells, suggesting that inhibition of cullin activity may impact the stability of phospho-S6K, or the phosphorylation of S6K at these residues (Figure 5D).

Finally, a phospho-peptide containing *p*-Ser9 and corresponding to reported PP2A-regulated protein GSK3β was identified with a fold change of 1.4 in dTAG-13-treated cells in comparison to DMSO-treated controls, but with a p value >0.05 (Table S2).^46^^,^^47^^,^^48^^,^^49^ By immunoblotting, the levels of *p*-Ser21/9 GSK3a/β were substantially increased in ^dTAG/dTAG^*PPP2CA* HEK293 cells treated with dTAG-13 over DMSO, while no changes were observed with dTAG-13 or DMSO treatment in WT HEK293 cells (Figure 5E). Co-treatment of dTAG-13-treated ^dTAG/dTAG^*PPP2CA* HEK293 cells with MLN4924, which rescues dTAG-PPP2CA degradation, caused a return in the levels of *p*-Ser21/9 GSK3a/β to those seen with DMSO treatment, while MLN4924 treatment alone had no effect (Figure 5E). These data suggest that, perhaps due to the vast number of changing phospho-peptides in response to dTAG-PPP2CA degradation and subsequent normalization, the phospho-peptide containing *p*-Ser9 GSK3β was not detected to be significantly enriched in our phospho-proteomic analysis. We also tested some other proteins that have been reported to be PP2A substrates or markers within PP2A-regulated pathways, including Akt and extracellular signal-regulated kinase 1/2 (ERK1/2),^75^^,^^76^^,^^77^ which were not significantly detected in our phospho-proteomic screen (Figure 5E). Only a modest increase in phosphorylation was observed for *p*-Thr308 Akt following dTAG-PPP2CA degradation (Figure 5E). This is potentially due to the fact that our experiments were performed in conditions where ERK1/2 and Akt signaling pathways were not stimulated with growth factors to induce high levels of phosphorylation of these target proteins.

### Further exploration of the impact of PPP2CA degradation

**Figure 6:**
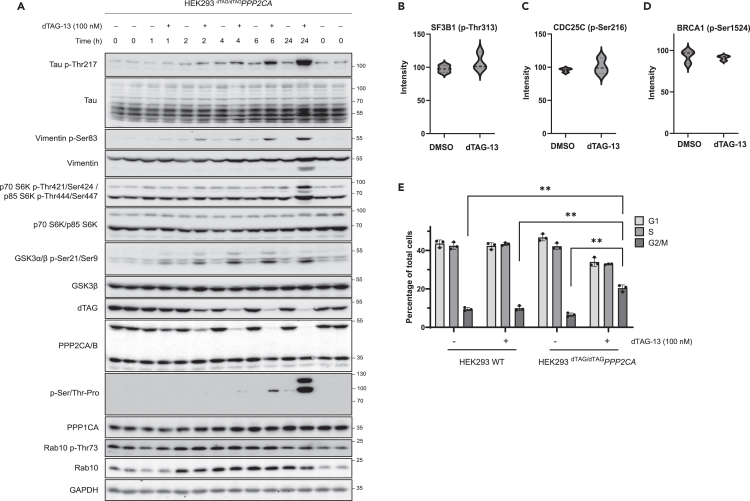
Further exploration of changes in phosphorylation and cell cycle distribution following PPP2CA degradation (A) ^dTAG/dTAG^*PPP2CA* HEK293 cells were treated with DMSO or dTAG-13 (100 nM) for the indicated durations before lysis. Samples were then resolved by SDS-PAGE and transferred to nitrocellulose membranes, which were analyzed by immunoblotting with the indicated antibodies. (B–D) Violin plots of some phospho-peptides identified by quantitative phospho-proteomic analysis that have been reported as PP1 substrates. Plots show no change in substrate phosphorylation upon dTAG-13 treatment, which mediates dTAG-PPP2CA degradation. (E) Cell cycle distribution was assessed by flow cytometric analysis using propidium iodide to quantify DNA content in WT and ^dTAG/dTAG^*PPP2CA* HEK293 cells following 24 h treatment with DMSO or dTAG-13 (100 nM). Data represent n = 3 and are shown as the mean ± SD percentage of cells in each cell cycle stage. Statistical analysis involved two-way analysis of variance (ANOVA) with Tukey’s multiple comparisons post-hoc test. Further related data available in Figure S8. Data are representative of three independent experiments.

To explore the rate of accumulation of these phospho-changes, we monitored the phospho-status of the above validated proteins over 24 h following PPP2CA degradation (Figure 6A). ^dTAG/dTAG^*PPP2CA* HEK293 cells were treated with DMSO or dTAG-13 for various durations prior to lysis. Subsequent immunoblotting revealed that, while small increases in the phosphorylation of tau, vimentin, S6K isoforms, and GSK3β were evident following 2 h dTAG-13 treatment, more substantial increases were apparent following 6 h or 24 h treatments. This requirement of a longer treatment duration to observe a marked accumulation of phospho-proteins may arise from the lack of specific stimulation in the experimental conditions employed. The dephosphorylation achieved by PPP2CA prior to degradation must be counteracted by kinase activity to replenish phosphorylation of the detected phospho-sites, with basal kinase activity potentially requiring more time to do so. Also, a small portion of the PPP2CA pool remains present at the 4 h and 6 h time points, which may enable a low level of dephosphorylation. Therefore, the 24 h time point selected for the phospho-proteomic analysis enables maximal PPP2CA degradation and sufficient time for the PPP2CA-depleted phosphorylation to be restored to detectable levels. In addition to monitoring PPP2CA-regulated proteins, we also monitored PP1 protein levels and the phosphorylation of reported PP1 substrates (Figures 6A–6D). Immunoblotting confirmed that PP1 levels were unaffected by dTAG13 treatment (Figure 6A). Furthermore, from our phospho-proteomic analysis, we observed that the phosphorylation of multiple reported PP1-regulated phospho-sites, *p*-Thr313 SF3B1/SAP155 (Figure 6B),^78^ *p*-Ser216 CDC25C (Figure 6C)^79^ and *p*-Ser1524 BRCA1 (Figure 6D)^80^ remained similar between 24 h DMSO and dTAG-13 treatments. As well as determining that PP1 protein levels and catalytic activity appear unaffected by dTAG-13 treatment, immunoblotting for phospho-Rab10, a reported PPM1H substrate,^81^ indicated no change in PPM1H activity upon dTAG-13 treatment for any of the explored durations (Figure 6A). Together, these findings indicate that the phospho-changes detected by our phospho-proteomic analysis are PP2A-dependent.

Given the well-established involvement of PP2A in mitosis and cell cycle control,^51^^,^^75^^,^^82^^,^^83^^,^^84^ as well as the implication of a significantly high percentage of our 2,204 proteins identified as putative PPP2CA substrates in the cell cycle (Figure 4A), we were interested to explore the impact of PPP2CA degradation on cell cycle distribution within the ^dTAG/dTAG^*PPP2CA* HEK293 cell population. WT and ^dTAG/dTAG^*PPP2CA* HEK293 cells were treated for 24 h with DMSO or dTAG-13 prior to staining with propidium iodide and subsequent flow cytometric analysis. A significantly higher portion of G2/M cells were identified in ^dTAG/dTAG^*PPP2CA* HEK293 cells treated with dTAG-13 in comparison to WT cells and DMSO-treated controls (Figures 6E, S8A, and S8B). Additionally, the width of the propidium iodide voltage pulse of the G2/M cells in ^dTAG/dTAG^*PPP2CA* HEK293 cells treated with dTAG-13 was higher than that in DMSO-treated ^dTAG/dTAG^*PPP2CA* HEK293 cells or WT controls, potentially indicating an increase in the number of mitotic cells or problems in mitosis (Figure S8C). These data corroborate the findings from the proliferation assay (Figure 1J) and the phospho-proteomic analysis (Figures 4 and S7), which demonstrate a critical role for PPP2CA in cell cycle control, mitosis, and cell proliferation.

## Discussion

In this study we combined CRISPR/Cas9 genome editing, PROTAC-mediated targeted protein degradation and unbiased phospho-proteomics to identify over two thousand proteins as putative substrates of PPP2CA, the major catalytic subunit of the PP2A holoenzyme complex. Some among these have been reported as PP2A substrates, but the vast majority are novel putative substrates, thus offering great potential to better understand substrate- and pathway-level phospho-regulation within cell signaling. Quantitative total proteome analysis upon targeted dTAG-PPP2CA degradation over 24 h revealed that PPP2CA was the only protein whose abundance was significantly altered (p < 0.05 and fold change greater than 2-fold), confirming the exquisitely selective nature of PROTAC-mediated protein degradation and also suggesting that inhibition of PPP2CA activity does not appear to impact the stability of its substrates. Despite the identification of >6,000 phospho-peptides as putative PPP2CA substrates upon its degradation over 24 h, surprisingly this did not cause substantial cytotoxicity over this time period. More predictably, sustained PPP2CA degradation over 48 h and 72 h caused a significant inhibition of cell proliferation.

Importantly, for some of the phospho-peptides that we identified as putative PPP2CA substrates by mass spectrometry, we were able to validate them by immunoblotting, suggesting our data to be robust. Given that the field of protein phosphatase research has lagged that of protein kinases, our approach has the potential to expedite the pace of research into phosphatases by identifying substrates in different biological settings, although care must be taken to ensure that the introduction of a degron tag does not compromise the function of the phosphatase in the first place. With selective degradation of dTAG-PPP2CA yielding increased phosphorylation of physiological PP2A substrate GSK3β, we demonstrate that incorporation of dTAG has not inhibited PPP2CA phosphatase activity in the ^dTAG/dTAG^*PPP2CA* HEK293 cell model used in this study.

*In silico* analysis of the phospho-peptide sequences enriched upon dTAG-PPP2CA degradation identified proline-directed *p*-Ser/p-Thr residues as key targets of PPP2CA. Other motif elements that we uncovered might provide insights into the residues upstream and downstream of the phospho-site that are or are not tolerated for PPP2CA-mediated dephosphorylation. Analysis of putative PP2A substrate proteins revealed their involvement in many key biological pathways, including spliceosome function, the cell cycle, RNA transport and surveillance, ubiquitin-mediated proteolysis, and DNA repair, which is consistent with the reported pleiotropic roles of PP2A holoenzyme complexes.^51^^,^^85^ Future studies will need to establish whether the PPP2CA-regulated dephosphorylation of individual proteins correlates with their functions in these biological processes and whether PPP2CA is directly or indirectly responsible for substrate dephosphorylation. Similarly, many enzymes, such as protein kinases and E3 ubiquitin ligases, were also identified as putative PPP2CA substrates, suggesting potential crosstalk between key regulators of cells signaling. For most of the identified phospho-sites, it is not known how phosphorylation affects the activities or behavior of these enzymes. We observed a significant association of putative PPP2CA substrates with breast cancer as well as numerous developmental disorders, such as micrognathism, developmental delay, microcephaly, mental retardation, congenital epicanthus, and neurodevelopmental disorders. Some putative PPP2CA substrates we identified that have previously been linked to neurodevelopmental disorders include RING finger protein 12 ( RNF12), OTU deubiquitinase 5 (OTUD5), methyl CpG binding protein 2 (MECP2), microcephalin 1 (MCPH1), fragile X mental retardation protein (FMRP), Ser/Arg-rich splicing factor protein kinase 2 (SRPK2), and budding uninhibited by benzimidazoles 1 (BUB1).^86^^,^^87^^,^^88^^,^^89^^,^^90^^,^^91^ RNF12 (also known as RLIM) is an E3 ligase that promotes the ubiquitin-mediated degradation of the transcription factor reduced expression 1 (REX1, also known as Zfp42), thus preventing transcription of neural genes.^92^^,^^93^ RNF12-dependent ubiquitination of REX1 was found to be stimulated following RNF12 phosphorylation by the SRPK (Ser/Arg-rich splicing factor (SRSF) protein kinase) kinase family.^86^ Interestingly, we observed enrichment of SRPK2-regulated phospho-sites upon dTAG-PPP2CA degradation, and even some phospho-sites on SRPK2 itself, potentially offering some insight into the association of hyper-phosphorylated hits with neurodevelopmental disorders. Previously, *de novo* mutations in *PPP2CA* and another PP2A subunit, PPP2R5D, have been identified in patients with intellectual disability and developmental delay.^94^^,^^95^ Furthermore, mice with conditional loss of PPP2CA in the central nervous system were reported to exhibit severe microcephaly, cortical atrophy and intellectual learning and memory defects,^85^^,^^96^ supporting an important role for PPP2CA in the context of development.

Our study demonstrates that targeted protein degradation and subsequent global phospho- and total-proteomic analysis is a robust approach to interrogate the potential substrates of protein phosphatases. PPP2CA is one of the catalytic subunits of the PP2A holoenzyme complex, with the other one being PPP2CB, which is 97% identical.^21^ Since our data suggests that PPP2CB was still expressed in ^dTAG/dTAG^*PPP2CA* HEK293 cells, it appears that PPP2CB was unable to fully compensate for the loss of PPP2CA and associated catalytic activity in these cells, potentially suggesting unique roles for these highly similar proteins. It would be interesting to identify PPP2CB substrates using a similar approach and to combine the degradation of PPP2CA and PPP2CB, to uncover unique and common PPP2CA and PPP2CB substrates, thus providing a full understanding of the extent of dephosphorylation conducted by PP2A holoenzyme complexes in cells.

### Limitations of the study

Although the introduction of the dTAG on PPP2CA did not affect some of its known substrates, it is possible that this might affect some other PPP2CA substrates. Given that we conducted the phospho-proteome analysis in ^dTAG/dTAG^*PPP2CA* HEK293 cells under cell culture conditions, another limitation of our study is the lack of biological or cell signaling context. However, the phospho-proteomics workflow and the cells we employed here can be applied to any cell signaling context for dissecting PPP2CA substrates in specific contexts. As with any phospho-proteomic study, the detection of putative substrates relies on the design and sensitivity of the approach, potentially meaning that the number of putative PP2A substrates we have identified is an underestimate of the true number of substrates. Indeed, while our high-throughput phospho-proteomics method successfully identified nearly 40,000 unique phospho-peptides, a significant portion of crucial regulatory phosphorylation events has proven elusive. This challenge arises because many sites are inaccessible or challenging to detect when subjected to trypsin digestion, which hinders MS-based investigations. Protein phosphorylation, particularly phosphorylation of multiple residues in close clusters, affects both proteolytic cleavage and ionization, thereby affecting detection by mass spectrometry. Utilizing multiple proteases in the phospho-proteomic analysis could be advantageous as it could improve throughput. Some of these issues could potentially account for other reported PPP2CA substrates not being significantly identified in our study, such as phospho-GSK3β, which we were able to validate by immunoblotting. Finally, the *in silico* analyses of the impact of putative PPP2CA substrates are solely based on the tools that we employed and have not been validated experimentally.

## STAR★Methods

### Key resources table

**Table:** 

REAGENT or RESOURCE	SOURCE	IDENTIFIER
**Antibodies**		

Rabbit polyclonal anti-Akt	Cell Signaling Technology	Cat# 9272S, RRID: AB_329827
Rabbit monoclonal anti-Akt *p*-Thr308	Cell Signaling Technology	Cat# 4056S
Rabbit monoclonal anti-Akt p-S473	Cell Signaling Technology	Cat# 4058
Rabbit polyclonal Cullin 2	Invitrogen	Cat# 51-1800
Sheep dTAG	MRC PPU Reagents & Services	Cat# DA179
Rabbit polyclonal ERK1/2	Cell Signaling Technology	Cat# 9102S
Rabbit polyclonal Erk1/2 (Thr202/Tyr204)	Cell Signaling Technology	Cat# 9101S
Rabbit polyclonal FKBP12	Abcam	Cat# ab24373
HRP-conjugated GAPDH Monoclonal antibody	ProteinTech	Cat# HRP-60004
GAPDH Monoclonal antibody	ProteinTech	Cat# 60004-1-Ig
GAPDH Monoclonal antibody	ProteinTech	Cat# 10494-1-AP
Rabbit monoclonal GSK-3β	Cell Signaling Technology	Cat# 9315
Rabbit polyclonal GSK-3α/β (Ser21/9)	Cell Signaling Technology	Cat# 9331S
Rabbit monoclonal PP2A A Subunit	Cell Signaling Technology	Cat# 2041
Mouse monoclonal PP2A B56α (PPP2R5A)	BD Transduction Laboratories	Cat# 61015
PPP2CA/B	MRC PPU Reagents & Services	Cat# S274B
Rabbit polyclonal p70 S6 Kinase	Cell Signaling Technology	Cat# 9202S
Rabbit polyclonal p70 S6 kinase *p*-Thr421/Ser424	Cell Signaling Technology	Cat# 9204
Rabbit polyclonal Phospho-(Ser/Thr) Phe	Cell Signaling Technology	Cat# 9631
Phospho-Ser/Thr-Pro MPM-2	Sigma	Cat# 05-368
Tau	MRC PPU Reagents & Services	Cat# S157B
Rabbit monoclonal Phospho-Tau (Thr217)	Cell Signaling Technology	Cat# 51625
Phosphotyrosine Mouse mAb (P-Tyr-100)	Cell Signaling Technology	Cat# 9411
Rabbit monoclonal Vimentin	Cell Signaling Technology	Cat# 5741S
Phospho-Vimentin (Ser83)	Cell Signaling Technology	Cat# 3878
Vinculin (E1E9V) XP® Rabbit mAb	Cell Signaling Technology	Cat# 13901

**Chemicals, peptides, and recombinant proteins**		

Triethylammonium bicarbonate buffer (TEAB)	Thermo Fisher Scientific	Cat # PI90114
Dithiothreitol	Sigma-Aldrich	Cat #D0632
Iodoacetamide (IAA)	Sigma-Aldrich	Cat #I6125
Trypsin	Promega	Cat #V5111
Lys-C	Wako	Cat #125–05061
Acetonitrile	JT Baker	Cat # 14650359
Fe(III)chloride, anhydrous	Sigma Aldrich	Cat#451649
Acclaim PepMap 100 2 cm trap column	Thermo Fisher Scientific	N/A
Acclaim PepMap 100C18 HPLC Column, 50 cm	Thermo Fisher Scientific	N/A
Urea	Thermo Fisher Scientific	Cat # 29,700
MLN4924	MRC PPU Reagents and Services	N/A
dTAG-13	MRC PPU Reagents and Services	N/A
PEI MAX – Transfection Grade Linear PEI Hydrochloride MW 40,000	Polysciences	Cat# 24765
Polybrene (Hexadimethrine bromide)	Sigma-Aldrich	Cat# 107689
MG132	Merck	474790-1MG
Dimethylsulphoxide	Sigma-Aldrich	Cat #D8418
Formic acid (FA)	Sigma-Aldrich	Cat #F0507
Trifluoroacetic acid	Sigma-Aldrich	Cat #T6508-100ML
PhosStop phosphatase inhibitor cocktail tablets	Roche	Cat#4906837001
Ni-NTA Superflow resin	Qiagen	Cat#30410

**Critical commercial assays**		

Pierce BCA Protein Assay Kit	Thermo Fisher Scientific	Cat#23225
TMTsixplex Isobaric Label Reagent Set	Thermo Fisher Scientific	Cat#90066
CellTox Green Assay	Promega	Cat. #G8742
CellTiter 96® AQueous One Solution Cell Proliferation Assay	Promega	Cat. #G3580

**Deposited data**		

Mapping the substrate landscape of protein phosphatase 2A catalytic subunit PPP2CA	This paper	PRIDE Project ID: PXD045779
Phosphoproteomics	This paper	https://curtainptm.proteo.info/#/7bbd6bee-d15f-415a-8ffe-da9f8bb91873
Proteomics	This paper	https://curtain.proteo.info/#/c16e75ef-879c-4e9a-9186-4bc4b5f28f0a
Data obtained in this study	This paper	Mendeley Data: https://data.mendeley.com/preview/ztpk2nwzg5?a=4d12b6c8-973d-40d7-b47e-e6288464a767

**Experimental models: Cell lines**		

Human: HEK293-FT	Invitrogen	Cat#R70007
Human: HEK293	ATCC	Cat. #CRL-1573

**Recombinant DNA**		

pCMV5-gag-pol	Cell Biolabs	Cat# RV-111
pCMV5-VSV-G	Cell Biolabs	Cat# RV-110
HA-dTAG-PPP2CA	MRC PPU Reagents & Services	DU77920
pCMV5D HA-PPP2CA	MRC PPU Reagents & Services	DU66347

**Software and algorithms**		

Proteome Discoverer platform (v2.4)	Thermo Fischer Scientific	NA
EnrichR	(Kuleshov et al., 2016)^63^	https://maayanlab.cloud/Enrichr/
Xcalibur	Thermo Fisher Scientific	Cat # OPTON-30965
GraphPad Prism v9.4.0	GraphPad	https://www.graphpad.com/scientific-software/prism/
KinMap beta tool	(Eid et al., 2017)^97^	http://www.kinhub.org/kinmap/index.html
Funrich (Version 3.1.3)	(Pathan et al., 2015)^65^	https://www.FunRich.org
STRING	(Snel et al., 2000)^66^	https://string-db.org/
FIMO (Find Individual Motif Occurrences)	(Grant et al., 2011)^98^	https://meme-suite.org/meme/tools/fimo
WebLogo	(Schneider and Stephens, 1990, Crooks et al., 2004)^52^^,^^53^	https://weblogo.berkeley.edu/

### Resource availability

#### Lead contact

Further information and requests for resources and reagents should be directed to and will be fulfilled by the Lead Contact: Gopal Sapkota (email: g.sapkota@dundee.ac.uk).

#### Materials availability

cDNA constructs are available to request from the MRC PPU Reagents and Services webpage (http://mrcppureagents.dundee.ac.uk) and the unique identifier (DU) numbers provide direct links to the cloning strategies and sequence details.

#### Data and code availability

• Original data have been deposited on Mendeley Data (https://data.mendeley.com/preview/ztpk2nwzg5?a=4d12b6c8-973d-40d7-b47e-e6288464a767) and are publicly available as of the date of publication. The link to access these data can also be found in the key resources table.
  All mass spectrometry data acquired from this study has been deposited in the PRIDE Archive, with the accession number PXD045779. Additionally, the data can be accessed via the following CurtainPTM^99^ links:
  For total proteomics (Table S1): https://curtain.proteo.info/#/c16e75ef-879c-4e9a-9186-4bc4b5f28f0a.
  For phospho-proteomics (Table S2): https://curtainptm.proteo.info/#/7bbd6bee-d15f-415a-8ffe-da9f8bb91873
• This paper does not report original code.
• Any additional information required to reanalyse the data reported in this paper is available from the lead contact upon request.

### Experimental model and study participant details

#### Cell lines

Aseptic technique that meets biological safety requirements was used for all procedures. HEK293 cells (ATCC, CRL-1573) are immortalized HEK cells derived from a female fetus. HEK293-FT cells (Invitrogen, Cat# R70007) are a clonal isolate of HEK293 cells transformed with the SV40 large T antigen. HEK293 and HEK293-FT cells were cultured in DMEM (Life Technologies) supplemented with 10% (v/v) fetal bovine serum (FBS, Thermo Fisher Scientific), 2 mM L-glutamine (Lonza), 100 U/mL penicillin (Lonza) and 0.1 mg/mL streptomycin (Lonza). Cells were maintained at 37°C with 5% CO_2_ in a water-saturated incubator and regularly tested for mycoplasma contamination. For passaging, trypsin/EDTA was used at 37°C to detach cells.

### Method details

#### Generation of cell lines using CRISPR/Cas9

The CRISPR/Cas9 genome editing system^42^ was used to generate HEK293 *PPP2CA* homozygous N-terminal dTAG KI (^dTAG/dTAG^*PPP2CA*) cells. HEK293 WT cells were transfected with vectors encoding a guide RNA (gRNA) targeting the *PPP2CA* exon 1 locus (DU69331, pX459 puromycin Cas9^D10A^ PPP2CA) (1 μg) and donor (DU69361, pMA PPP2CA Nter GFP IRES2 FKBP12^F36V^) (3 μg), as well as polyethylenimine (PEI, 1 mg/ml). 16 h post-transfection, selection with 1 μg/mL puromycin (Sigma-Aldrich) was carried out for 48 h. The transfection process was replicated (without a further round of selection). Cells were sorted by flow cytometry and single cells were plated in individual wells of 96-well plates. Viable clones were expanded, and integration of dTAG at the target locus was verified by Western blotting, polymerase chain reaction (PCR) amplification and genomic sequencing of the targeted locus.

#### Plasmids

The following vectors were used for transient transfection or for production of retroviral vectors: pBABED (puromycin) HA-dTAG-PPP2CA (DU77920) and pCMV5D HA-PPP2CA (DU66347). All constructs were sequence-verified by the DNA Sequencing Service, University of Dundee (http://www.dnaseq.co.uk). These constructs are available to request from the MRC PPU Reagents and Services webpage (http://mrcppureagents.dundee.ac.uk) and the unique identifier (DU) numbers provide direct links to the cloning strategies and sequence details.

#### Transient transfection of cells

Transient transfection of cDNA plasmids was performed on sub-confluent (60–70%) cells in 15 cm dishes. 3 μg cDNA, 30 μL PEI, and 2 mL Opti-MEM were vortexed for 20 s, incubated at room temperature for 20 min and added dropwise to cells. Cells were incubated in the transfection mix for 24 h prior to lysis.

#### Retroviral generation of stable cell lines

Retroviral pBABED-puromycin vectors encoding the desired construct (6 μg) were co-transfected with pCMV5-gag-pol (3.2 μg) and pCMV5-VSV-G (2.8 μg) (Cell Biolabs) into a 10 cm diameter dish of ∼70% confluent HEK293-FT cells. Plasmids were added to 1 mL Opti-MEM medium in addition to 24 μL of 1 mg/mL PEI before gentle mixing and incubation at room temperature for 20 min. The transfection mix was then added dropwise to HEK293-FT cells. 16 h post-transfection, fresh medium was added to the cells. After 24 h, the retroviral medium was collected and passed through 0.45 μm sterile syringe filters. Target cells (∼60% confluent) were transduced with the optimized titer of the retroviral medium diluted in fresh medium (typically 1:1) containing 8 μg/mL polybrene (Sigma-Aldrich) for 24 h. Then the cells were placed in fresh medium containing the appropriate concentration of antibiotic to select cells which had integrated the construct with a control non-transduced plate put under selection in parallel. A pool of transduced cells was utilized for subsequent experiments following complete death of the control plate.

#### Treatment of cells with compounds

The following chemicals were added to cell media using the treatment durations and concentrations indicated in figure legends: dimethylsulphoxide (DMSO) (Sigma-Aldrich), dTAG-13 (MRC PPU Reagents and Services), MLN4924 (MRC PPU Reagents and Services) and MG132 (Merck). Unless stated otherwise, an equivalent volume of DMSO was used.

#### Cell lysis and immunoprecipitation

Cells were harvested by washing twice with phosphate-buffered saline (PBS) and scraping into ice-cold lysis buffer (50 mM Tris-HCl pH 7.5, 0.27 M sucrose, 150 mM NaCl, 1 mM ethylene glycol-bis(β-aminoethyl ether)-N,N,N′,N′-tetraacetic acid (EGTA), 1 mM ethylenediaminetetraacetic acid (EDTA), 1 mM sodium orthovanadate, 10 mM sodium β-glycerophosphate, 50 mM sodium fluoride, 5 mM sodium pyrophosphate and 1% NP-40) supplemented with 1× cOmplete protease inhibitor cocktail (PIC). Lysates were incubated for 10 min on ice before clarification by centrifugation at 17,000 G for 20 min at 4°C. The Bradford assay was used to determine protein concentration and enable normalisation between samples.

For immunoprecipitation (IP), cells were lysed as above and a 1 mL solution containing 2 mg protein was then subjected to immunoprecipitation using 25 μL of a 50/50 (v/v) slurry made using HA-frankenbody resin slurry (MRC Reagents and Services) with lysis buffer (without phosphatase inhibitors) supplemented with PIC. 75 μL was removed for input samples and added to 15 μL 6X SDS sample buffer. IP samples were then incubated for 2 h with rotation at 4°C. Flow-through samples were then removed (75 μL). IP samples were eluted in 75 μL 2X SDS sample buffer and 15 μL of IP and input samples were subjected to immunoblotting. IP:input ratio is ∼10:1.

#### SDS-PAGE and Western blotting

Cell lysates containing equal amounts of protein (20 μg) were resolved by SDS-PAGE and transferred to nitrocellulose membrane. Membranes were blocked in 5% (w/v) non-fat milk (Marvel) in tris-buffered saline, 0.2% Tween 20 (TBS-T) (50 mM Tris–HCl pH 7.5, 150 mM NaCl, 0.2% Tween 20) and incubated overnight at 4°C in 5% (w/v) bovine serum albumin (BSA)/TBS-T or 5% (w/v) milk/TBS-T with the appropriate primary antibodies. Primary antibodies used at indicated dilutions include: anti-Akt (9272, CST, 1:1000), anti-Akt *p*-Thr308 (4056S, CST, 1:1000), anti-Akt *p*-Ser473 (4058, CST, 1:1000), anti-Cullin 2 (51–1800, Invitrogen, 1:1000), anti-dTAG (DA179, MRC PPU Reagents and Services, 1 μg/mL), anti-ERK1/2 (9102S, CST, 1:1000), anti-ERK1/2 p-Thr202/Tyr204 (9101S, CST, 1:1000), anti-FKBP12 (used to detect dTAG, also known as FKBP12^F36V^) (ab24373, Abcam, 1:1000), anti-GAPDH-HRP (HRP-60004, ProteinTech, 1:30000), anti-GAPDH (10494-1AP, ProteinTech, 1:30,000), anti-GAPDH (60004-1-Ig, ProteinTech, 1:30,000), anti GSK3-β (9315S, CST, 1:1000), anti GSK3-α/β *p*-Ser21/Ser9 (9331S, CST, 1:1000), anti-PP2A A subunit (PPP2R1A) (2041, CST, 1:1000), anti-PP2A B56α (PPP2R5A) (610615, BD Transduction Laboratories, 1:1000), anti-PPP2CA/B (S274B, MRC PPU Reagents & Services, 1 μg/mL), anti-p70 S6 kinase (9202S, CST, 1:1000), anti-p70 S6 kinase *p*-Thr421/Ser424 (9204, CST, 1:1000), anti-*p*-Ser/Thr-Phe (9631, CST, 1:1000), anti-*p*-Ser/Thr-Pro MPM-2 (05–368, Sigma, 1:1000), anti-Tau (S157B, MRC PPU Reagents and Services, 1 μg/mL), anti-Tau *p*-Thr217 (51625, CST, 1:1000), anti-*p*-Tyr (9411, CST, 1:1000), anti-Vimentin (5741S, CST, 1:1000), anti-Vimentin *p*-Ser83 (3878, CST, 1:1000), anti-Vinculin (13901S, CST, 1:1000).

Membranes were subsequently washed with TBS-T and incubated with horseradish peroxidase (HRP)- or IRDye 800CW-conjugated secondary antibody for 1 h at room temperature. HRP-coupled secondary antibodies used include: goat anti-rabbit-IgG (7074, CST, 1:5000), rabbit anti-sheep-IgG (31480, Thermo Fisher Scientific, 1:5000), goat anti-mouse-IgG (31430, Thermo Fisher Scientific, 1:5000). IRDye 800CW-coupled secondary antibodies used include: IRDye 800CW Donkey anti-Rabbit IgG (H + L) (926–32213, Licor, 1:5000). After further washing, signal detection was performed using enhanced chemiluminescence (ECL) (Merck) for HRP-conjugated secondaries and ChemiDoc MP System (Bio-Rad). Image Lab (Version 6.0.1) (Bio-Rad) was used to analyze protein bands by densitometry.

#### Cell cytotoxicity assay

CellTox Green Assay (Promega, Cat. #G8742) was used to assess the cytotoxicity of dTAG-13-mediated dTAG-PPP2CA degradation in ^dTAG/dTAG^*PPP2CA* HEK293 cells as well as in control WT HEK293 cells (12–48 h, 100 nM dTAG-13 or DMSO control). The fluorescent signal produced by the CellTox Green dye, upon selective binding to the DNA of cells with impaired membrane integrity, is proportional to cytotoxicity. Fluorescence was measured using ex: 480 nm em: 530 nm by a PHERAstar FS plate reader before subtracting blank measurements (made using wells containing medium only, no cells) and normalising to DMSO treatment. MG132 treatment (40 μM) was included as a positive control at each time point. Data was analyzed using Excel (Microsoft) and GraphPad Prism software (Version 8).

#### Cell proliferation assay

The CellTiter 96 AQ_ueous_ One Solution Cell Proliferation Assay (Promega, Cat. #G3580) was used to assess the impact of dTAG-13-mediated dTAG-PPP2CA degradation on cell proliferation in ^dTAG/dTAG^*PPP2CA* HEK293 cells, as well as in control WT HEK293 cells. Cells were treated with dTAG-13 (100 nM) or DMSO for 24, 48 or 72 h before incubation with the CellTiter 96 AQ_ueous_ One Solution Reagent. The reagent contains a tetrazolium compound [3-(4,5-dimethylthiazol-2-yl)-5-(3-carboxymethoxyphenyl)-2-(4-sulfophenyl)-2H-tetrazolium, inner salt; MTS(a)] that is bioreduced by cells to form a colored formazan product. Formation of the formazan product can be measured by absorbance at 490 nm. A media-only “blank” control was included for each time point, which was subtracted from sample absorbance values of the same time point. Absorbance of dTAG-13-treated samples was then normalized to DMSO treatment. MG132 treatment (40 μM) was included as a positive control for each cell line and each time point. Three technical replicates were included for each condition, with three separate biological replicates also being conducted. Data was analyzed using Excel (Microsoft) and GraphPad Prism software (Version 8).

#### Flow cytometry analysis of cell cycle distribution

Wild-type (WT) and ^dTAG/dTAG^*PPP2CA* HEK293 cells were treated for 24 h with DMSO or dTAG-13 prior to processing for flow cytometric analysis as follows. Following treatments, cells were trypsinized, gently centrifuged and resuspended in 1 mL PBS +1% FBS (v/v). Cells were pelleted and resuspended in 1 mL ice-cold 90% methanol while vortexing to prevent clumps. After a 30 min incubation at room temperature, samples were adjusted to contain 5 × 10^5^ cells. Following two washes with PBS +1% FBS (v/v), cells were pelleted and resuspended in 300 μL staining buffer (50 μg/mL propidium iodide and 50 μg/mL RNase A in PBS +1% FBS (v/v)). Samples were incubated at room temperature for 20 min protected from light. Samples were then analyzed by flow cytometry using an LSR Fortessa flow cytometer and data was subsequently analyzed using FlowJo software. Doublets and clumps were excluded from the analysis by gating on the basis of propidium iodide (PI)-Area vs. -Width. Cell cycle analysis was performed using PI-Area measurements using the Watson Pragmatic Model to determine each phase of the cell cycle.

#### Mass spectrometry global proteome and phospho-proteome analysis

Cells were lysed in urea lysis buffer (8 M urea, 50 mM Triethylammonium bicarbonate buffer (TEAB) pH 8.0, supplemented with 1 tablet of cOmplete protease inhibitors per 25 mL lysis buffer and 1 tablet of PhosSTOP phosphatase inhibitors per 10 mL lysis buffer) by Bioruptor sonication for 15 cycles at 30 s intervals in LoBind Eppendorf tubes. Lysates were clarified by centrifugation for 20 min at 13,000 G at 4°C. Protein concentration was estimated using the Pierce bicinchoninic acid method. Equal protein from each condition was reduced with 5 mM dithiothreitol (DTT) at room temperature for 30 min and alkylated with 20 mM iodoacetamide (IAA) in the dark at room temperature for 15 min. Samples were then digested with Lys-C (1:100) at 30°C for 4 h. Samples were then diluted with 50 mM Triethylammonium bicarbonate buffer (TEAB) to a urea concentration of 1.5 M and were then digested with trypsin (1:20) at 30°C for 16 h. The digestion was quenched with the addition of trifluoroacetic acid (TFA) to give a final concentration of 1% TFA (v/v) and samples were desalted on 200 mg Sep-Pak C_18_ cartridges (Waters). For Sep-Pak clean-up, the following solvents were prepared fresh: activation solvent (100% (v/v) acetonitrile (ACN)); Solvent-1 (0.1% (v/v) TFA); Solvent-2 (0.1% (v/v) formic acid (FA)); Solvent-3 (50% (v/v) ACN in 0.1% (v/v) FA). Sep-Pak cartridges were equilibrated with 5 mL 100% ACN, followed by 5 mL 50% ACN, 0.1% FA and finally with 5 mL 0.1% TFA twice. Samples were then loaded onto the equilibrated C_18_ cartridges, washed with 5 mL 0.1% TFA four times, followed by washing with 5 mL 0.1% FA. Samples were then eluted with 6 mL 50% ACN, 0.1% FA. Desalted samples were then dried to completeness in a SpeedVac concentrator.

Peptides were resuspended in 50 mM TEAB and labeled using TMT labels as per the manufacturer’s instructions. TMT labels were resuspended in anhydrous acetonitrile, added to assigned samples and incubated for 1 h at room temperature. Peptides derived from DMSO-treated controls were labeled with TMT labels 126, 127N and 127C, while peptides derived from dTAG-13-treated cells were labeled with 128N, 128C and 129N. Following label check by LC-MS/MS, the labeling reaction was quenched with 5% hydroxylamine for 15 min at room temperature. Labeled peptides from each condition were pooled together and dried.

Pooled peptides were separated by basic reverse phase chromatography fractionation on a C_18_, 250 × 4.6 mm column, 5 μm, XBridge (Waters, Milford, MA) with flow rate at 500 μL/min with two buffers: buffer A (10 mM ammonium formate, pH 10) and buffer B (80% ACN, 10 mM ammonium formate, pH 10). Peptides were resuspended in 100 μL of buffer A (10 mM ammonium formate, pH10) and resolved on a C_18_ reverse phase column by applying a non-linear gradient of 7–40%. A total of 96 fractions were collected and concentrated into 24 fractions. 90% was used for immobilized metal affinity chromatography (IMAC)-based phospho-peptide enrichment and the remaining 10% for proteomic analysis. Each concentrated fraction was then dried by SpeedVac.

IMAC beads were prepared from Ni-NTA (nitrilotriacetic acid) superflow agarose beads that were stripped of nickel with 100 mM EDTA and incubated in an aqueous solution of 10 mM iron (III) chloride (FeCl_3_). Dried peptide fractions were reconstituted to a concentration of 0.5 μg/μL in 80% ACN/0.1% TFA. Peptide mixtures were enriched for phosphorylated peptides with 10 μL IMAC beads for 30 min with end-to-end rotation. Enriched IMAC beads were loaded on Empore C_18_ silica packed stage tips. Stage tips were equilibrated with methanol followed by 50% ACN/0.1% FA then 1% FA. The beads with enriched peptides were loaded onto C_18_ stage tips and washed with 80% ACN/0.1% TFA. Phosphorylated peptides were eluted from IMAC beads with 500 mM dibasic sodium phosphate, pH 7.0. These peptides were washed with 1% FA before elution using 50% acetonitrile in 0.1% FA. The peptides were then dried by SpeedVac and stored at −20°C until mass spectrometry analysis. Enriched phospho-peptides and peptides were analyzed on an Orbitrap Fusion Tribrid mass spectrometer interfaced with Dionex Ultimate 3000 nanoflow liquid chromatography system.

For phospho-peptide analysis, peptides were enriched on a trap column (Acclaim PepMap100 C_18_, 100 μm × 2 cm, 5 μm, 100 Å) and separated on an analytical column (Acclaim PepMap RSLC C_18_, 75 μm × 50 cm, 2 μm, 100 Å) at a flow rate of 300 nL/min using a step gradient of 5–7% solvent B (90% ACN/0.1% FA) for the first 10 min, followed by 7–30% up to 105 min. The total run time was set to 140 min. The mass spectrometer was operated in a data-dependent acquisition mode. A survey full scan MS (from m/z 375–1500) was acquired in the Orbitrap at a resolution of 120,000 at 200 m/z. The automatic gain control (AGC) target for MS1 was set as 2 × 10^5^ and ion filling time set at 50 ms. The most intense ions with charge state ≥2 were isolated and fragmented using higher collision dissociation (HCD) fragmentation, with 38% normalized collision energy, and detected at a mass resolution of 60,000 at 200 m/z. The isolation window was set at 0.7. The AGC target for MS2 was set as 5 × 10^4^ and ion filling time set at 118 ms, while dynamic exclusion was set for 60 s.

For proteomic analysis, peptides were separated on an analytical column (Acclaim PepMap RSLC C_18_, 75 μm × 50 cm, 2 μm, 100 Å) at a flow rate of 300 nL/min, using a step gradient of 5–7% solvent B (90% ACN/0.1% FA) for the first 10 min, followed by 7–25% up to 70 min and 25–35% up to 70–85 min. The total run time was set to 100 min. The mass spectrometer was operated in a data-dependent acquisition mode in SPS MS3 (FT-IT-HCD-FT-HCD) method. A survey full scan MS (from m/z 400–1400) was acquired in the Orbitrap at a resolution of 120,000 at 200 m/z. The AGC target for MS1 was set as 4 × 10^5^ and ion filling time as 50 ms. The precursor ions for MS2 were isolated using a Quadrupole mass filter at a 0.7 Da isolation width, fragmented using a normalized 32% HCD of ion routing multipole and analyzed using ion trap. The top 10 MS2 fragment ions in a subsequent scan were isolated and fragmented using HCD at a 65% normalized collision energy and analyzed using an Orbitrap mass analyser at a 50,000 resolution, in the scan range of 100–500 m/z.

The proteomics raw data were searched using SEQUEST HT search engines with Proteome Discoverer 2.4 (Thermo Fisher Scientific). The following parameters were used for searches: Precursor mass tolerance 10 ppm, Fragment mass tolerance 0.1, Enzyme: trypsin, Mis-cleavage: −2, Fixed modification: carbamidomethylation of cysteine residues and TMT of lysine and N-terminal, Dynamic modification: oxidation of methionine. The data were filtered for 1% PSM, peptide and protein level FDR. Only unique peptides were selected for the quantification.

Phospho-peptide-enriched fractions from each replicate were searched against the Uniprot protein database using the SEQUEST HT search engines with Proteome Discoverer 2.4 (Thermo Fisher Scientific). A 10 plex TMT reporter ion workflow was loaded and the following search parameters were used: trypsin protease was selected; two missed cleavages were allowed; oxidation of Met and phosphorylation of Ser/Thr/Tyr were set as variable modifications; and carbamidomethylation of Cys, TMT of lysine and N-terminal were set as fixed modifications. The mass error tolerance for MS1 and MS2 (10 ppm and 0.02 Da) was used. The data were filtered for 1% PSM, peptide and protein level FDR. For identification of the phospho-site probability, the ptmRS node was used.

### Quantification and statistical analysis

For Western blot data: Western blot densitometry was measured using Image Lab and adjusted relative densities were calculated using Excel (Microsoft). All statistical analyses were performed and graphs were generated using GraphPad Prism software (Version 8). Statistical details including the exact value of n and any statistical tests performed are stated in the figure legends. Graphs display the mean ± standard deviation (SD) of 3 independent experiments, unless stated otherwise in figure legend.

For proteomic data: The SEQUEST output protein group text files were processed using the Perseus software suite.^100^ The data was filtered for any proteins identified only by site, common contaminants and reverse hits and proteins identified with single unique peptides. The reporter ion intensities were log2 transformed and the data was normalized by the median for each sample independently. Student-T test was performed and permutation-based false discovery rate of 5% was applied to identify the differentially enriched and significant protein groups. For cluster analysis, the multiple T test ANOVA was carried out with Benjamin Hochberg correction false discovery rate (FDR) of 5%. Significant changes were classified by p < 0.05 and a fold change greater than either 1.5-fold for phospho-proteomic analysis or 2-fold for total proteomic analysis.

#### Bioinformatics analysis

For motif analysis, 16-mer peptides containing the phosphorylated residue at the center were extracted from the MaxQuant output file and used for motif analysis using WebLogo (available at: https://weblogo.berkeley.edu/).^52^^,^^53^ For the comparison of our data with known PPP2CA substrates, we searched our identified substrates against PPP2CA substrates listed on the DEPOD database - the human DEPhOsphorylation Database (available at: http://depod.bioss.uni-freiburg.de/).^59^ The KinMap beta tool^97^ (available at: http://www.kinhub.org/kinmap/index.html) was used to build the kinome map. We plotted the list of kinases identified in our dataset as potential PPP2CA substrates and highlighted them on the kinome map. To identify the upstream kinases whose substrates are overrepresented among our identified putative PPP2CA substrates, KEA3 was carried out (available at: https://maayanlab.cloud/kea3/#results).^101^ We used Funrich (Version 3.1.3) (https://www.FunRich.org)^65^ and EnrichR (available at: https://maayanlab.cloud/Enrichr)^62^^,^^63^ to conduct the KEGG pathway analysis, uncover associations with biological processes, molecular function, localization, protein domain architecture and diseases. Further protein–protein interaction network analysis for the putative PPP2CA substrates was carried out using STRING (available at: https://string-db.org/).^66^ The proteins that did not show any connection with the network were removed for clarity. To identify proteins containing the PP2A-specific SLiM, the LSPIxE motif was scanned across the human proteome using FIMO (available at: https://meme-suite.org/meme/tools/fimo).^98^

## Supplemental information

Document S1. Figures S1–S8

Table S1. Proteomics data, related to Figure 2 Full list of total proteomics data.

Table S2. Phospho-proteomics data, related to Figure 2 Full list of phospho-proteomics data, including separate sheets for Significant, Hyper-phosphorylated and Hypo-phosphorylated hits.

Table S3. Results from bioinformatic analyses, related to Figure 3 and Figure 4 sFull lists of results from different bioinformatic analyses conducted in this study using the hyper-phosphorylated hits are detailed in separate sheets, including for KEGG pathway analysis, biological processes, molecular functions, protein domains, subcellular localization, disease associations and comparisons with DEPOD database.
