# Supplementary material for: Mapping the substrate landscape of protein phosphatase 2A catalytic subunit PPP2CA
Source: iScience. 2024 Feb 19;27(3):109302. doi: 10.1016/j.isci.2024.109302 (PMC10915630; doi:10.1016/j.isci.2024.109302)
Supplement: Document S1. Figures S1–S8 [file mmc1.pdf]

iScience, Volume *27*

Supplemental information

Mapping the substrate landscape of protein phosphatase 2A catalytic subunit PPP2CA

Abigail Brewer, Gajanan Sathe, Billie E. Pflug, Rosemary G. Clarke, Thomas J. Macartney, and Gopal P. Sapkota

<u>**Supplementary Materials**</u>

SUPPLEMENTARY FIGURES

Figure S1

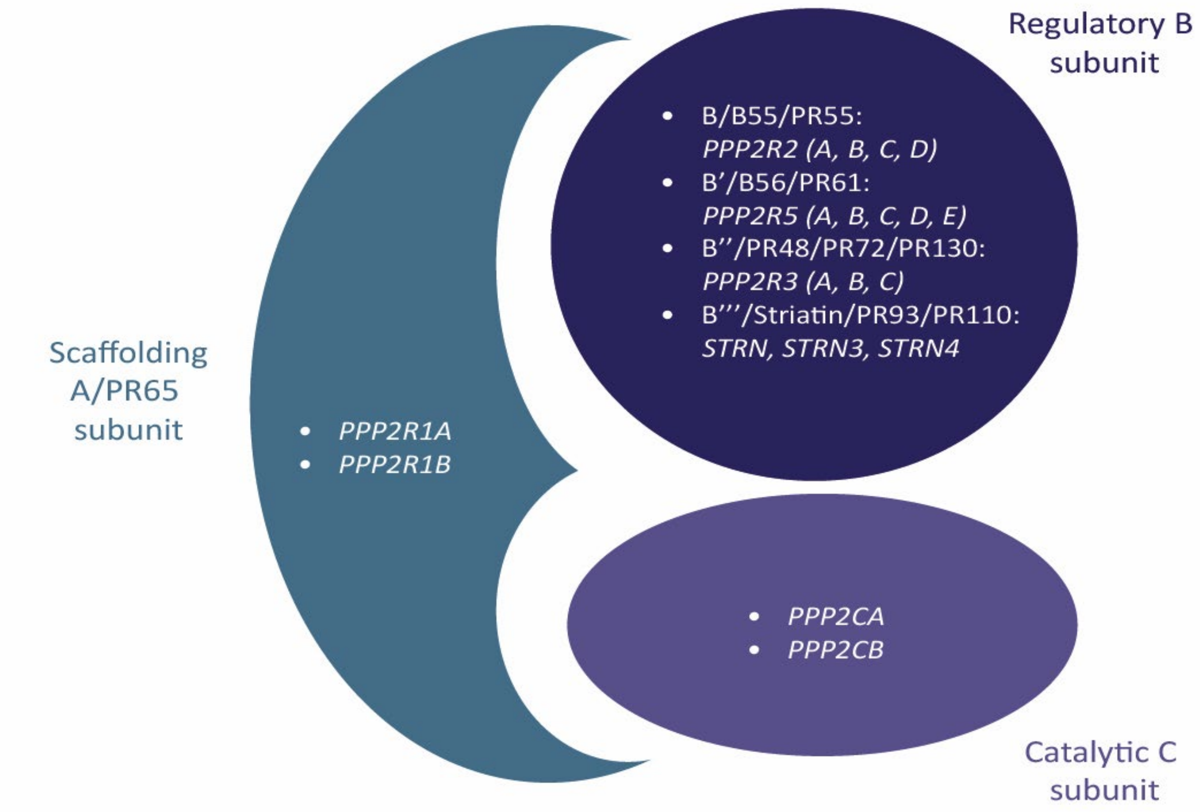

Figure S1. Human PP2A phosphatase holoenzyme structure with genes encoding different subunit isoforms. Related to Figure 1. PP2A holoenzyme complex detailing genes encoding distinct isoforms of the scaffolding A, regulatory B and catalytic C subunits.

Figure S2

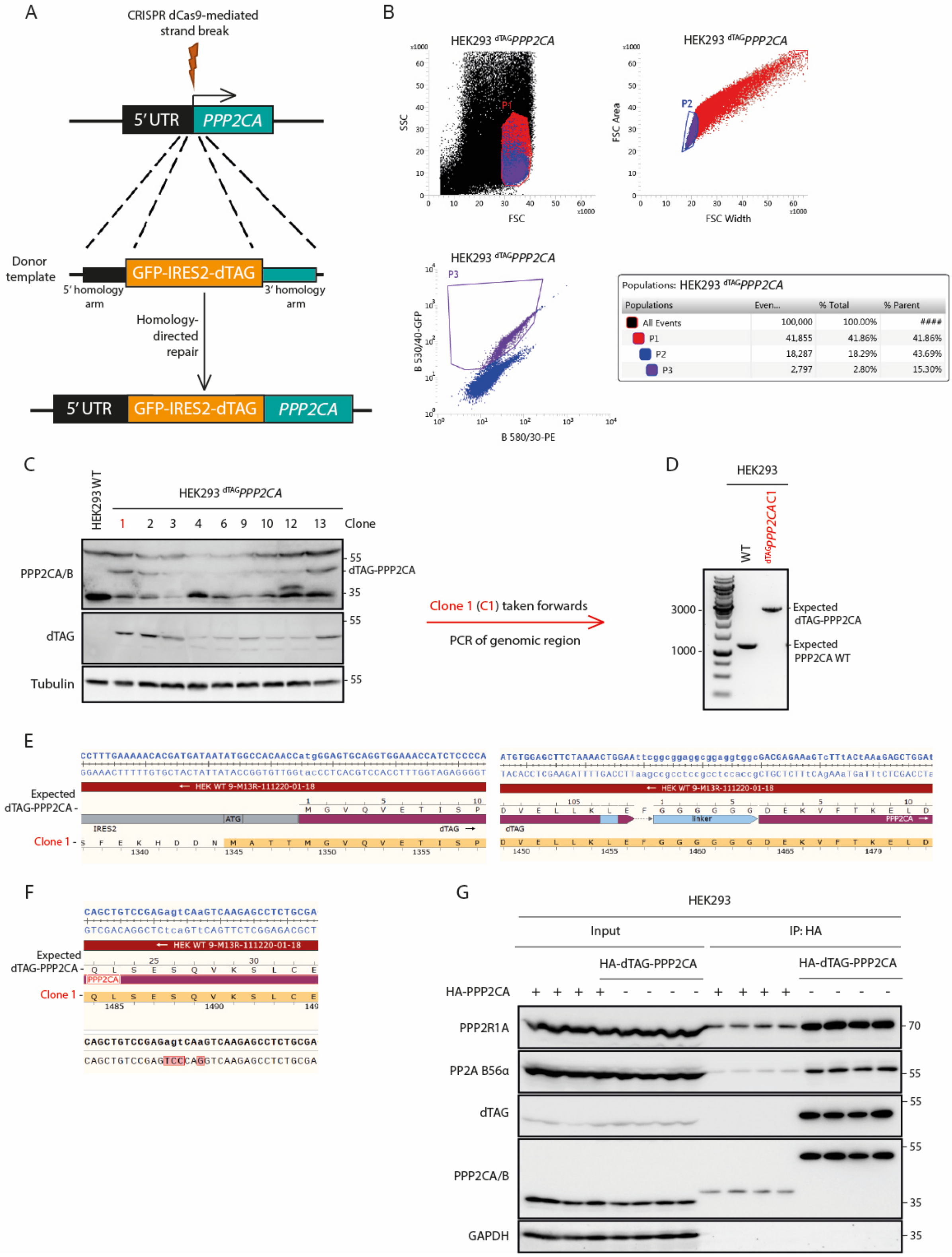

Figure S2. Confirmation of $^{dTAG/dTAG}$*PPP2CA* knock-in in HEK293 cells. Related to Figure 1.

A. Depiction of CRISPR strategy to introduce dTAG at N-terminus of *PPP2CA*.

B. Fluorescence-activated cell sorting (FACS) analysis of HEK293 GFP-IRES2-dTAG-PPP2CA cells identified 15.30% of cells as being GFP-positive.

C. Initial immunoblot screen of isolated single cell clones from 24-well plates identified clone 1, among others, as potential homozygous for $^{dTAG/dTAG}$*PPP2CA* HEK293 knock-in. Clones were lysed and samples were then resolved by SDS-PAGE and transferred to nitrocellulose membranes, which were analysed by immunoblotting with the indicated antibodies. Anti-FKBP12 antibody was used to detect dTAG (also known as FKBP12^{F36V}).

D. Confirmation of knock-in in HEK293 cells by polymerase chain reaction (PCR), using *PPP2CA* forward (Fw) and reverse (Rev) primers that bind upstream of the 5'-UTR region and downstream of the start of the *PPP2CA* gene to give a PCR product of ~2.8 kbp for dTAG-PPP2CA or a product of ~1.2 kbp for wild type (WT) PPP2CA. Clone 1 was identified as containing homozygous knock-in of $^{dTAG/dTAG}$*PPP2CA*.

E. Genomic DNA sequencing and subsequent alignment with the expected DNA sequence at IRES2-dTAG and dTAG-*PPP2CA* boundaries confirmed successful knock-in. Alignment conducted using SnapGene.

F. Sequence alignment of genomic DNA extract from $^{dTAG/dTAG}$*PPP2CA* HEK293 Clone 1 cells identified expected silent mutations at residues 26 and 27 that were introduced on the donor to block recognition by the guide RNAs. Alignment conducted using SnapGene.

G. Confirmation that dTAG-PPP2CA can interact with other PP2A subunits to form a holoenzyme complex. HEK293 cells stable expressing HA-dTAG-PPP2CA or transiently transfected to express HA-PPP2CA were subjected to anti-HA immunoprecipitation. Cell lysates and anti-HA IP samples were processed for immunoblot as in **A** and probed with antibodies recognising the PP2A A (PPP2R1A) and B56α (PPP2R5A) subunits.

Figure S3

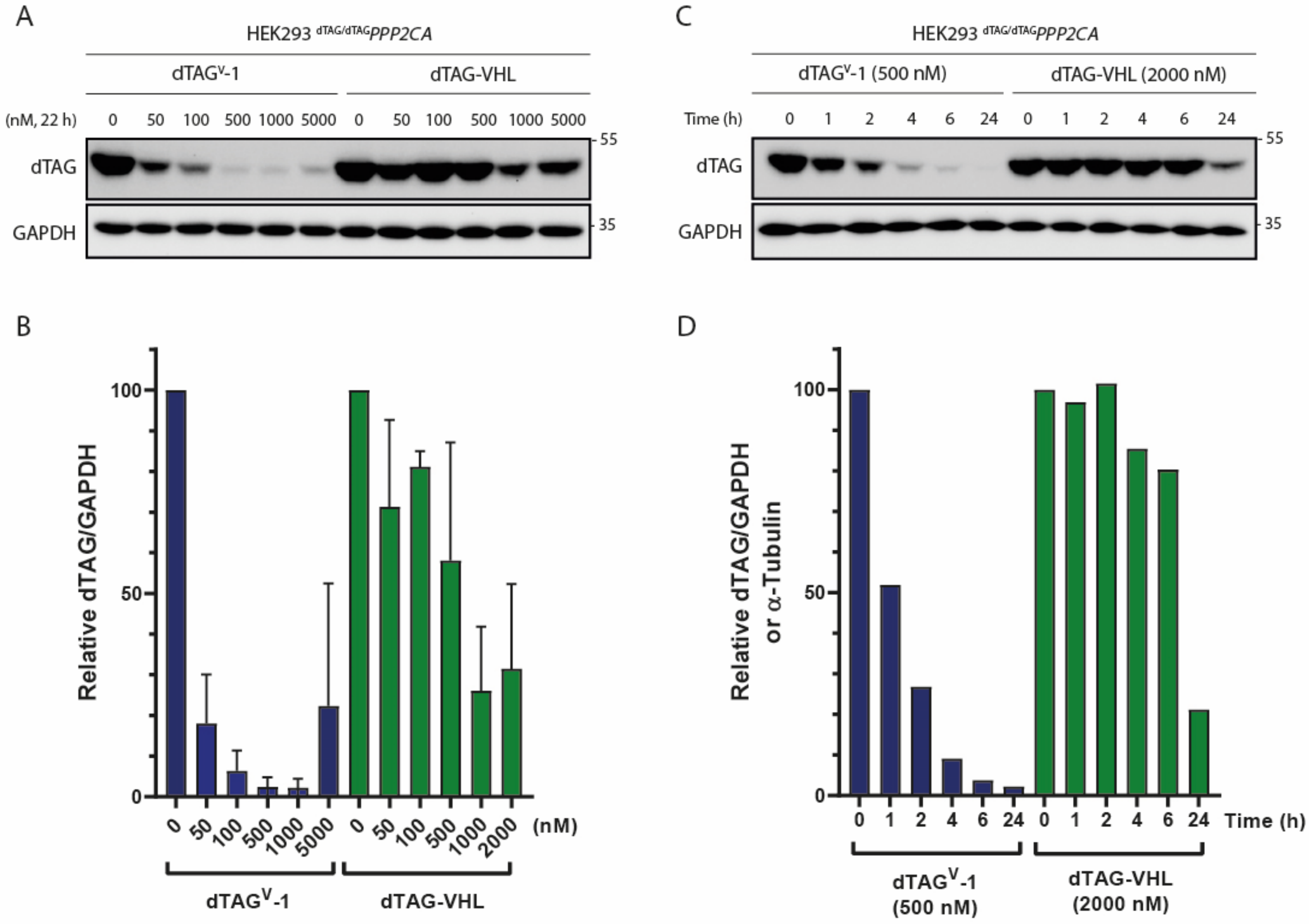

Figure S3. Degradation of dTAG-^{V}1 and dTAG-VHL PROTACs. Related to Figure 1.

A-B. $^{dTAG/dTAG}$*PPP2CA* HEK293 cells were treated with the indicated concentrations of dTAG^{V}-1, dTAG-VHL or DMSO for 22 h prior to lysis. Samples were then resolved by SDS-PAGE and transferred to nitrocellulose membranes, which were analysed by immunoblotting with the indicated antibodies. Anti-FKBP12 antibody was used here to detect dTAG (also known as FKBP12^{F36V}). Three biological replicates are quantified in **B**, where mean values are displayed for normalised (dTAG/GAPDH), relative to DMSO-treated control samples. Error bars represent standard deviation.

C-D. $^{dTAG/dTAG}$*PPP2CA* HEK293 cells were treated with 500 nM dTAG^{V}-1 or 2000 nM dTAG-VHL for the indicated durations prior to lysis and immunoblot analysis. Quantification of two biological replicates is shown in **D**, with mean values displayed, relative to 0 h treatment.

Figure S4

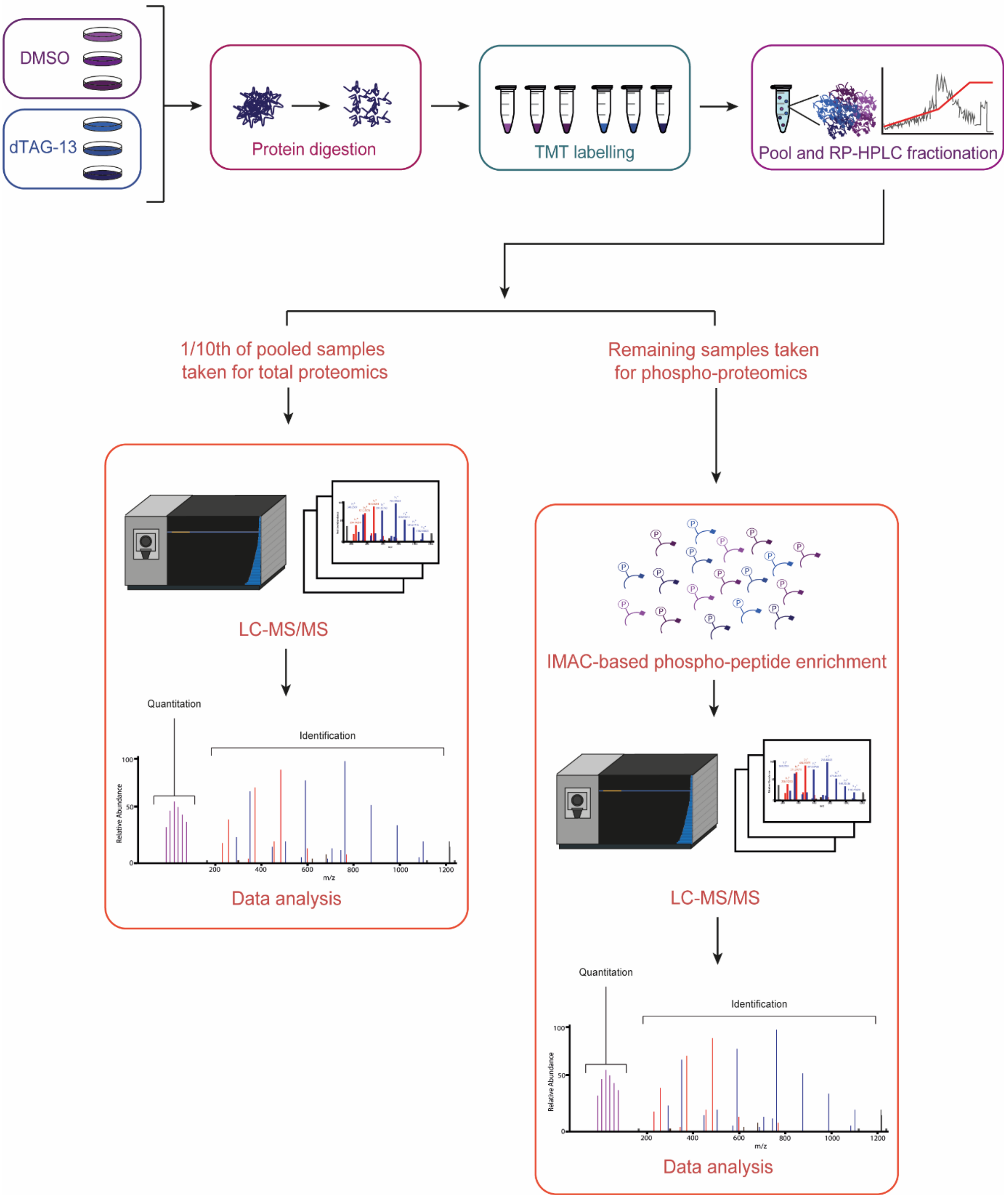

Figure S4. Global total- and phospho-proteomic approach to explore substrate landscape of PPP2CA. Related to Figure 2. Workflow of total- and phospho-proteomic sample preparation and analysis using $^{dTAG/dTAG}$*PPP2CA* HEK293 cells treated with dTAG-13 (100 nM) or DMSO for 24 h.

Figure S5

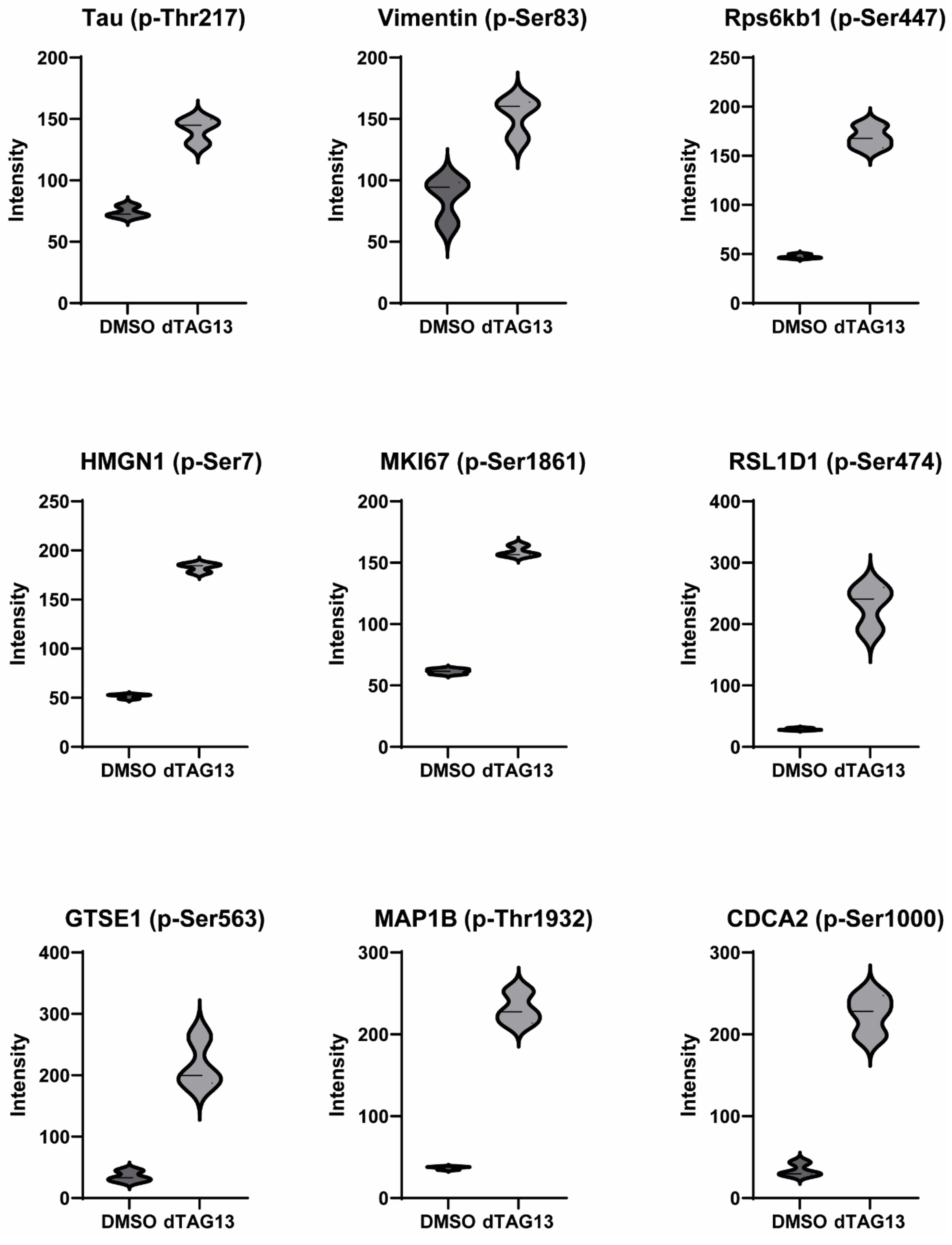

Figure S5. Violin plots of selected phospho-peptides identified from quantitative phospho-proteomic analysis. Related to Figure 2 and Figure 5. Some phospho-peptides shown here have been validated by immunoblotting in Figure 5.

Figure S6

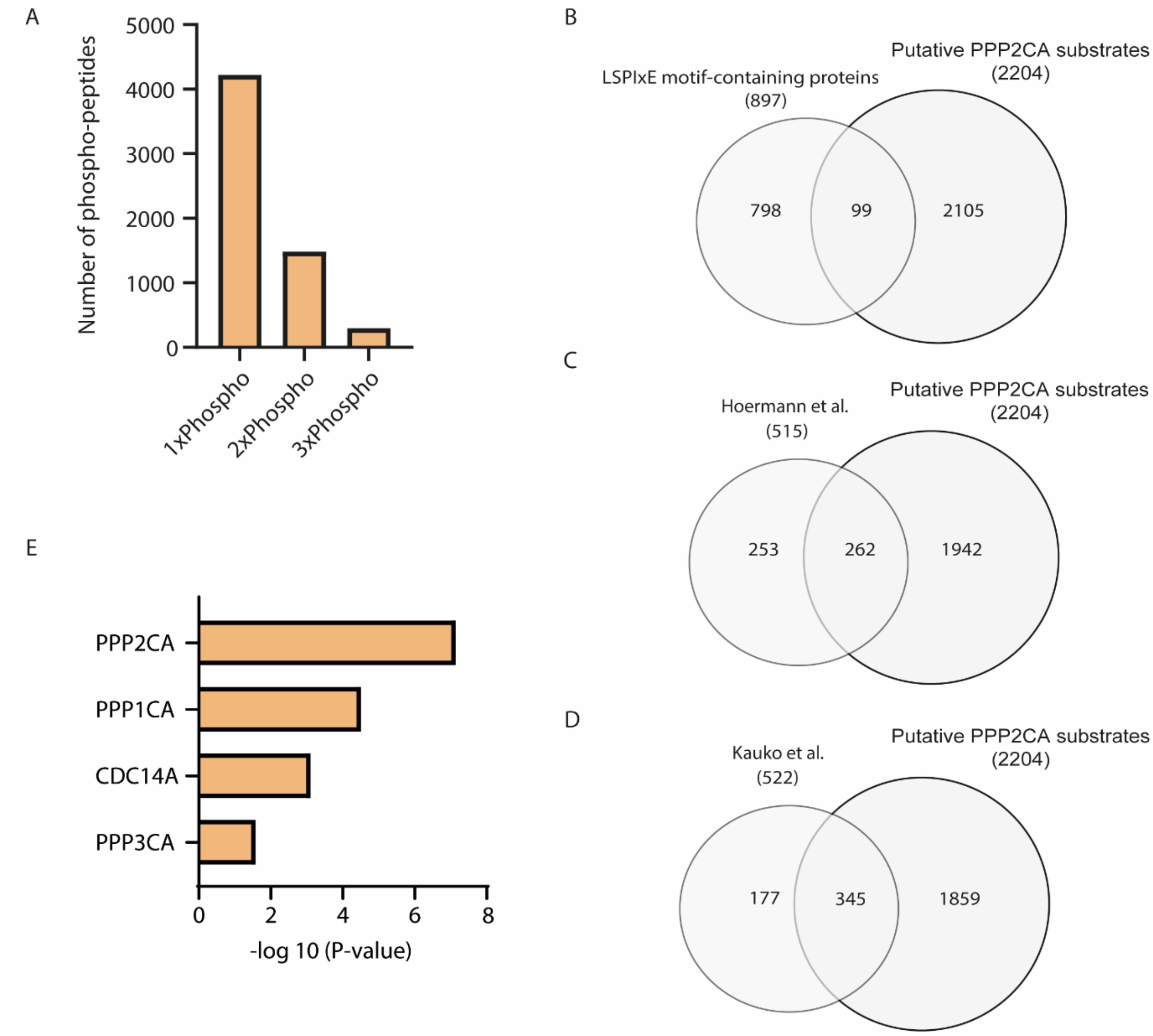

Figure S6. Analysis of putative PPP2CA substrates. Related to Figure 3.

A. The prevalence of mono-, di- and tri-phosphorylated peptides amongst the phospho-peptides we identified to be enriched upon dTAG-PPP2CA degradation.

B. PP2A-B56α-specific SLiM LSPIxE was scanned across the human proteome using Find Individual Motif Occurrences (FIMO) (available at: https://meme-suite.org/meme/tools/fimo). This identified 897 unique proteins containing the LSPIxE SLiM. We compared these with our identified putative PPP2CA substrates, with common proteins displayed in the Venn diagram.

C. Comparison between PPP2CA substrates identified by Hoermann et al. (2020) and putative PPP2CA substrates identified in our study.

D. Comparison between PPP2CA substrates identified by Kauko et al. (2020) and putative PPP2CA substrates identified in our study.

E. Using Enrichr, putative PPP2CA substrates identified in our study were compared with the DEPOD database to uncover phosphatases the database predicted to be most likely to dephosphorylate these substrates (available at: https://maayanlab.cloud/Enrichr).

Figure S7

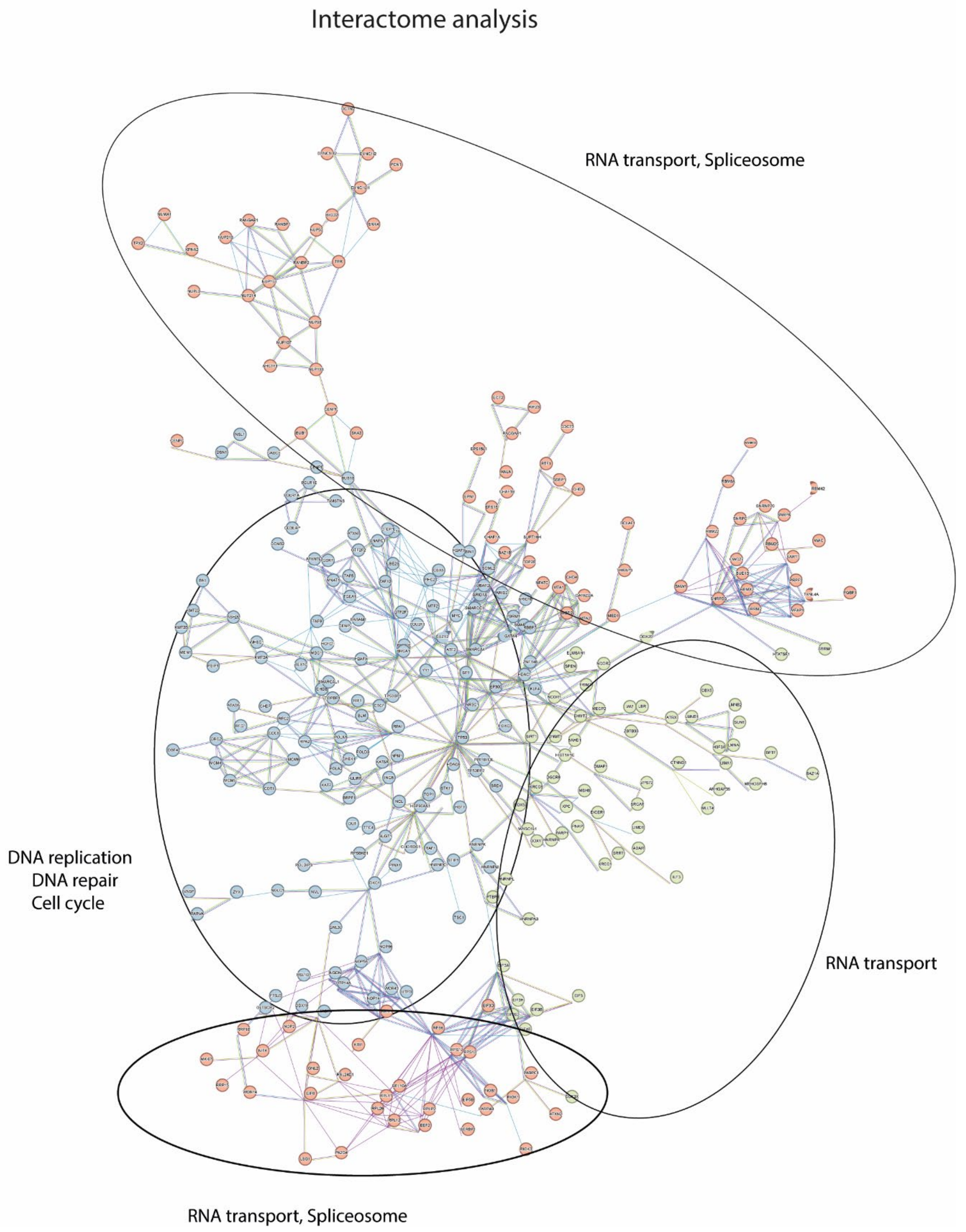

Figure S7. Network analysis of phospho-proteins identified as putative PPP2CA substrates from the phospho-proteomic analysis. Related to Figure 4. Four major enriched pathways are circled and presented. The colour of the nodes indicates the biological process that the proteins in the network are involved in.

Figure S8

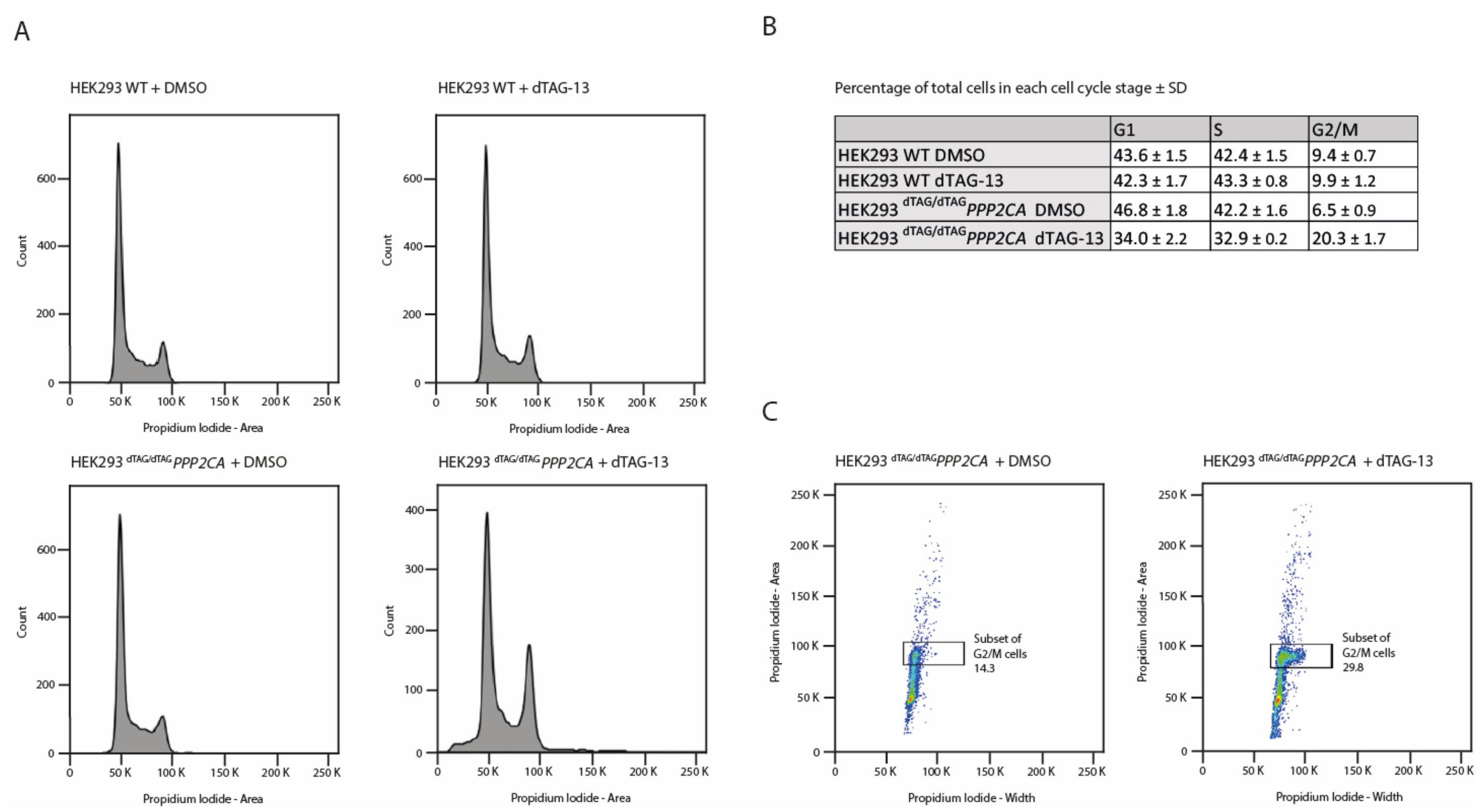

	G1	S	G2/M
HEK293 WT DMSO	43.6 ± 1.5	42.4 ± 1.5	9.4 ± 0.7
HEK293 WT dTAG-13	42.3 ± 1.7	43.3 ± 0.8	9.9 ± 1.2
HEK293 $^{dTAG/dTAG}$*PPP2CA* DMSO	46.8 ± 1.8	42.2 ± 1.6	6.5 ± 0.9
HEK293 $^{dTAG/dTAG}$*PPP2CA* dTAG-13	34.0 ± 2.2	32.9 ± 0.2	20.3 ± 1.7

Figure S8. Flow cytometry analysis of cell cycle distribution following dTAG-13 treatment. Related to Figure 6.

Cell cycle distribution was assessed by flow cytometry, using propidium iodide to quantify DNA content in WT and $^{dTAG/dTAG}$*PPP2CA* HEK293 cells following 24 h treatment with DMSO or dTAG-13 (100 nM). Data represent three independent experiments.

A. Representative flow cytometry results are shown, which were obtained following propidium iodide staining of cell populations. From these data, the distribution of cells across G1, S and G2/M cell cycle phases was determined, as displayed in Figure 6E.

B. Percentages of cells attributed to each cell cycle stage for the populations tested. These data were used to generate Figure 6E.

C. A subset of G2/M phase cells, as defined by propidium iodide staining, is indicated for $^{dTAG/dTAG}$*PPP2CA* HEK293 cells treated with DMSO or dTAG-13 to demonstrate the difference in Propidium Iodide-Width between the two conditions.

Supplementary tables

Table S1. Related to Figure 2. Full list of total proteomics data.

Table S2. Related to Figure 2. Full list of phospho-proteomics data, including separate sheets for Significant, Hyper-phosphorylated and Hypo-phosphorylated hits.

Table S3. Related to Figure 3 and Figure 4. Full lists of results from different bioinformatic analyses conducted in this study using the hyper-phosphorylated hits are detailed in separate sheets, including for KEGG pathway analysis, biological processes, molecular functions, protein domains, subcellular localisation, disease associations and comparisons with DEPOD database.
